# Atomic Scaled Depth Correlation to the Oxygen Reduction Reaction Performance of Single Atom Ni Alloy to the NiO_2_ Supported Pd Nanocrystal

**DOI:** 10.1002/advs.202207109

**Published:** 2023-02-08

**Authors:** Haolin Li, Sheng Dai, Yawei Wu, Qi Dong, Jianjun Chen, Hsin‐Yi Tiffany Chen, Alice Hu, Jyh‐Pin Chou, Tsan‐Yao Chen

**Affiliations:** ^1^ School of Materials Science and Engineering Zhejiang Sci‐Tech University Hangzhou 310018 China; ^2^ Department of Engineering and System Science National Tsing Hua University Hsinchu 300044 Taiwan; ^3^ Department of Mechanical Engineering City University of Hong Kong Hong Kong SAR 999077 China; ^4^ School of Chemistry and Molecular Engineering East China University of Science and Technology Shanghai 200234 China; ^5^ Department of Electrical Engineering Tsinghua University Beijing 100084 China; ^6^ Department of Materials Science and Engineering City University of Hong Kong Hong Kong SAR 999077 China; ^7^ Department of Physics National Changhua University of Education Changhua 50007 Taiwan; ^8^ Hierarchical Green‐Energy Materials (Hi‐GEM) Research Centre National Cheng Kung University Tainan 70101 Taiwan; ^9^ Department of Materials Science and Engineering National Taiwan University of Science and Technology Taipei 10617 Taiwan

**Keywords:** bimetallic catalysts, DFT calculations, fuel cell, oxygen reduction reaction

## Abstract

This study demonstrates the intercalation of single‐atom Ni (Ni^SA^) substantially reduces the reaction activity of Ni oxide supported Pd nanoparticle (NiO_2_/Pd) in the oxygen reduction reaction (ORR). The results indicate the transition states kinetically consolidate the adsorption energy for the chemisorbed O and OH— species on the ORR activity. Notably, the NiO_2_/Ni^1^/Pd performs the optimum ORR behavior with the lowest barrier of 0.49 eV and moderate second‐step barrier of 0.30 eV consequently confirming its utmost ORR performance. Through the stepwise cross‐level demonstrations, a structure–*E*_ads_–Δ*E* correspondence for the proposed NiO_2_/Ni*^n^*/Pd systems is established. Most importantly, such a correspondence reveals that the electronic structure of heterogeneous catalysts can be significantly differed by the segregation of atomic clusters in different dimensions and locations. Besides, the doping‐depth effect exploration of the Ni^SA^ in the NiO_2_/Pd structure intrinsically elucidates that the Ni atom doping in the subsurface induces the most fruitful Ni^SA^/Pd^ML^ synergy combining the electronic and strain effects to optimize the ORR, whereas this desired synergy diminishes at high Pd coverages. Overall, the results not only rationalize the variation in the redox properties but most importantly provides a precision evaluation of the process window for optimizing the configuration and composition of bimetallic catalysts in practical experiments.

## Graphical abstract

A diagram illustrating how charge transfer occurs between NiO_2_, interfacial Ni atoms, outer Pd‐shell atoms, and the surface species involved in the oxygen reduction reaction in the NiO_2_/Ni_1_/Pd system. Using small amounts of inexpensive nickel at the interface with costly subsurface‐Pd layer creates a unique local synergy, leading to improved reactivity and selectivity in the catalysts.

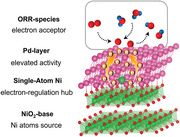

## 1 Introduction

Fuel cells (FCs) are widely acknowledged as the most promising renewable energy technologies owing to their cost‐efficient, zero‐emission, and environment‐friendly essences fitting perfectly with the global rising issue of carbon‐neutral.^[^ ^1^, ^2^, ^3^ ^]^ The heterogeneous catalyst for oxygen reduction reaction (ORR) performance is the key bottleneck that takes the largest overpotential loss, the lowest reaction kinetics and the highest production cost among all components in FCs, hereby attracting intensive resources in academic research.^[^ ^4^, ^5^, ^6^, ^7^, ^8^ ^]^ The acidic FCs have earlier penetrated into the FCs commercial market due to their relatively matured materials and engineering processes ahead of other kinds.^[^ ^9^ ^]^ However, the redox dynamics of cathodic catalyst in such type of FC is lower than that of alkaline electrolyte ones (AFCs). In the AFCs, the ORR is inherently more efficient and less passivated than in acidic conditions.^[^ ^10^, ^11^ ^]^ The corresponding reaction coordinates are proceeding via a four‐electron (4e^−^) route that includes two substeps, the “O_2_ dissociation” and subsequent “O* (adsorbed atomic oxygen) hydrogenation.” Such a scenario enables the natural advantages of transition metals for facilitating the first O_2_ dissociation step (commonly regarded as the rate determine step (i.e., RDS)^[^ ^12^, ^13^, ^14^ ^]^:

Substep 1: O_2_ dissociation

(1.1) $$O_{2}\to O^{*}+O^{*}$$

Substep 2: O* hydrogenation

(1.2) $$2O^{*}+2H_{2}O+4e^{-}\to 2{\mathrm{OH}}^{-}+2{\mathrm{OH}}^{-}$$

consequently, shining light on the possibility of a substantially reduced noble metal usage in AFCs cathode.

Until now, precious metal platinum (Pt) is commonly recognized as the second‐to‐none for AFCs due to its well verified first‐class ORR activity compared with other single‐crystal metals in both theoretical and practical approaches,^[^ ^15^, ^16^ ^]^ nonetheless, its persistent earth scarcity as well as the resulting high overall cost, critically limiting the commercial applications of the functional materials with high Pt loading.^[^ ^17^ ^]^ Hence, the screen for low‐ or even non‐Pt electrocatalyst with high performance is imperative to access large‐scale industrialization for the FCs.^[^ ^18^, ^19^ ^]^ Palladium (Pd) catalyst, which is an important member of the “Pt clan” transition metals, features the high‐performing ORR that second only to Pt catalyst according to its electrochemical activity,^[^ ^20^, ^21^ ^]^ theoretical volcano‐plot position^[^ ^22^, ^23^ ^]^ and relatively more resource‐abundant.^[^ ^24^, ^25^ ^]^ Although the material price is increasing, the recycle technologies and feasibility of Pd are relatively accessible as compared to those of Pt. In addition, some experimental evidences demonstrated that the Pd NCs perform higher ORR performance than Pt ones in AFCs.^[^ ^26^, ^27^ ^]^ Meanwhile, to further improve the ORR activity and reduce the material cost, effective attempts by alloying with transition metals (e.g., Mo, Ni, Cu)^[^ ^19^, ^28^, ^29^ ^]^ or metal oxides (e.g., TiO_2_, MoS_2_, or ZrO_2_)^[^ ^30^, ^31^, ^32^ ^]^ had been achieved. Nickel oxide (NiO_2_), due to its large lattice mismatch and a low cost to the noble metal, is regarded as a prospective candidate for foreign support combined with highly active metal to synergistically explore the electrocatalyst's commercial potential.^[^ ^33^, ^34^ ^]^ Even so, inadequate activity augmentation and unwished structure disintegration is still difficult to be solved due to the inevitable disordered structures of general alloying modification.^[^ ^35^ ^]^

Aiming at screening the potential structures or combinations at nano‐ or atomic‐scale for Pd‐based catalyst with the superhigh ORR, a well‐ordered core/shell‐like structure, which is theoretically derived based on the *d*‐band model theory developed by Nørskov and Hammer et al.^[^ ^36^, ^37^, ^38^ ^]^ and the optimum catalyst idea of the Sabatier principle,^[^ ^39^ ^]^ is considered to be an effective design strategy to controllably improve the ORR activity as well as the structural stability by introducing the synergy of ligand effect and strain effect into catalysts.^[^ ^40^, ^41^, ^42^ ^]^ Apart from those structure optimization engineering (i.e., size, alloy, bifunctional, ligand, and lattice strain),^[^ ^43^, ^44^, ^45^, ^46^ ^]^ the geometric configuration and the local confinement of the active atomic clusters in the metal and metal oxide interface raises a quantum leap on the kinetics of redox reaction in the heterogeneous catalyst surface. Such a scenario had been demonstrated in ORR by decorating atomic clusters of various sizes and species in the metallic nanoparticles.^[^ ^43^, ^47^, ^48^ ^]^ In these scenarios, the changes in the adsorption energy (*E*_ads_) for the intermediate species (i.e., O* and OH), the *d*‐band center (*ε*_d_), projected DOS, and the charge density distribution are intensively studied. These results all revealing the facilitation of ORR performance by the combination effects of electron localization and the gradient distribution for the *E*_ads_ around the decorated atomic clusters. However little had been solved on the location and distribution of the atomic catalysts with extreme dimension control from several atoms to a single‐atom (SA) level in metal catalysts (i.e., single‐atom catalyst, SAC).

To address all the above‐mentioned issues and obtain the NCs with a series of those desirable characteristics, meanwhile considering the unavoidable heteroatomic intermix occurring during fast wet chemical crystallization experimentally, we propose a NiO_2_ supported Pd (NiO_2_^core^/Pd^shell^) with different levels of Ni atoms intercalated in the shell Pd layer for evaluating the ORR activity by using density functional theory (DFT) calculation. With the increasing number of Ni atom(s) from few‐atom to many‐atom, the models are named the “NiO_2_/Ni*^n^*/Pd” catalyst systems (*n* = 1, 2, 3, 4, 7, 10, 13, 1ML, and 2ML). Our multilevel evaluating scenario, including the intermediates adsorption energies, *d*‐band center, PDOS, and physical charge transfer calculations, reveals the focal role of the interfacial intercalated Ni^SA^ as the “electron‐regulation hub” in the NiO_2_/Ni^1^/Pd ternary system. Consequently, it synergistically boosts the best‐performing ORR behavior with the optimum reaction barriers of the two subpaths compared to the rest systems, which undoubtedly outperforms the benchmark Pd enormously. Meanwhile, this excited synergistic effect improves the potential instability of the heterogeneous structures via enhancing the internal interface bonding. Our deep‐going investigation of the Ni^SA^ doping depth elucidates the subsurface‐doping of Ni^SA^ (i.e., the interlayer of three‐layer Pd, Ni^SA^‐2^nd^) endowing the NiO_2_/Pd structure the maximum improvement in ORR by raising the most remarkable Ni^SA^/Pd^ML^ synergy combining the electronic influence and strain effect, compared to the bottom‐layer and top‐layer doping ones. By cross‐referencing the results, we establish a structure‒*E*_ads_‒Δ*E* correspondence for our proposed NiO_2_/Ni*^n^*/Pd systems concerning the ORR. Corresponding results demonstrate that the electronic structure of heterogeneous catalysts is significantly controlled by the dimension of atomic clusters. In addition, the location is the strongest effective factor among existing structure parameters in the reaction coordinate of the decorated NCs in ORR. For further clarification, the above statements will be discussed comprehensively in the following sections.

## 2 Computational Details

First‐principles calculations are performed within the Vienna ab initio simulation package (VASP)^[^ ^49^, ^50^, ^51^ ^]^ based on DFT with the projector augmented wave (PAW)^[^ ^52^ ^]^ method. To describe the exchange and correlation interaction, the Perdew–Burke–Ernzerhof (PBE)^[^ ^53^ ^]^ functional under the generalized gradient approximation (GGA)^[^ ^54^ ^]^ is used. The total energy of bulk shows convergence with 20 × 20 × 20 Monkhorst‐Pack *k*‐point sampling in the Brillouin zone and 420 eV cut‐off kinetic energy. A 4 × 4 supercell with six atomic layers was used to mimic the Pd(111) and the NiO_2_/Ni*^n^*/Pd catalysts in a close‐packed FCC(111) stacking, which is consistent with our earlier work.^[^ ^55^, ^56^, ^57^ ^]^ The NiO_2_/Ni*^n^*/Pd (*n* = 1, 2, 3, 4, 7, 10, 13, 1ML, and 2ML) systems were made up of three layers of Pd(111) on the two trilayers of NiO_2_ with nine distinct Ni*^n^* substitution on Pd atom (superscript *n* denotes the number of doped Ni atoms). Herein, a 15 Å vacuum space was used to avoid the interaction between periodic slabs. All the involved adsorbates and the uppermost three (four) layers of the slabs were fully relaxed, while the bottom two layers of the NiO_2_ (Pd) were fixed. A grid of 5 × 5 × 1 *k*‐point sampling was used for the slab supercell geometry optimization and transition‐state (TS) calculations, while a refined 7 × 7 × 1 *k*‐point sampling was implemented for precise electronic calculations (e.g., the DOS and charge analysis). The calculated optimized lattice constant of the Pd(111) and proposed NiO_2_/Ni*^n^*/Pd surface model are 3.94 and 3.95 Å under PBE functional, respectively. Total energy sensitivity was examined converged for the lattice constant, *k*‐point sampling, kinetic energy cut‐off, slab thickness, and vacuum space. In this thesis, the formation energy *E*_f_ is expressed as the following equation

(2.1) $$E_{f}=\left(E_{sys}-\sum_{i}n_{i}\mu_{i}\right)\;\;/\;N$$

here, *n_i_* and *µ_i_* are the number of atoms and chemical potential of the species *i*, respectively; *E*_sys_ is the total energy of the system. The chemical potential was calculated using the bulk energy per atom. While *N* denotes the total number of atoms that correspond to *E*_sys_. The adsorption energy *E*_ads_ is used to measure the strength of the bond between the slab and its adsorbate, which is defined by the equation

(2.2) $$E_{\mathrm{ads}}=E_{\mathrm{sys}}-E_{*}-E_{\mathrm{surf}}$$

here, the energy of the overall system is *E*_sys_, the energy of the adsorbed species is E_*_, and the energy of the surface is *E*_surf_.

The TS was determined using the climbing image nudged elastic band (CI‐NEB) calculation through the VTST code,^[^ ^58^, ^59^ ^]^ and the reaction barriers related to the energy difference between the initial state (IS) and the TS. In this work, the reaction barrier ∆*E* is calculated by the following equation

(2.3) $$\Delta E_{n}=TS_{n}-IS_{n}$$

here, *n* = 1 or 2, which corresponds to the two different ORR substages on different surface models, i.e., “1” means the “O_2_ dissociation” stage, and “2” denotes the “O* hydrogenation” stage. The *E*_f_ and *E*_ads_ after optimization were used to determine the IS and final‐state (FS) model configurations. Bader charge population was conducted on the basis of the grid‐based methodology developed by Henkelman et al., which was primarily utilized to measure the charge around an atom.^[^ ^60^, ^61^ ^]^ The isosurface of the charge density difference was presented to qualitatively study the charge transfer situation around embedded Ni atoms in the NiO_2_/Ni*^n^*/Pd systems as follows

(2.4) $$\Delta \rho =\rho_{\mathrm{total}}-\rho_{\mathrm{slab}}-\rho_{\mathrm{Ni}}$$

here *ρ*_total_ denotes the entire system's total charge density; *ρ*_slab_ means the charge density of the slab without decorated Pt; and *ρ*_Ni_ is the charge density of the individual Ni atoms fixed in its original position of the slabs, respectively.

## 3 Results and Discussion

### 3.1 Determination of Slab Model Configurations

**Figure 1:**
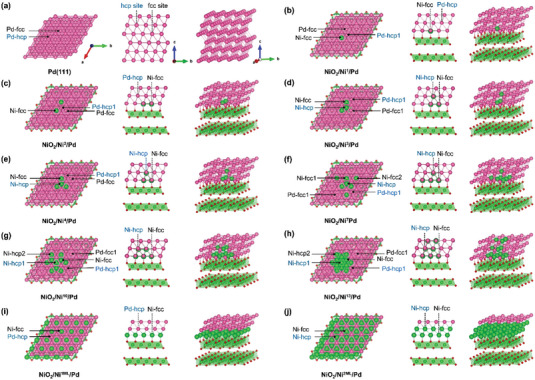
Top, side, and oblique views for the model catalysts of interest. a) Pure Pd(111), b) NiO_2_/Ni^1^/Pd, c) NiO_2_/Ni^2^/Pd, d) NiO_2_/Ni^3^/Pd, e) NiO_2_/Ni^4^/Pd, f) NiO_2_/Ni^7^/Pd, g) NiO_2_/Ni^10^/Pd, h) NiO_2_/Ni^13^/Pd, i) NiO_2_/Ni^1ML^/Pd, and j) NiO_2_/Ni^2ML^/Pd. For clarity, the key threefold hcp and fcc sites for O* of each surface model are marked by blue and black arrows; the pink, green, and red spheres represent Pd, Ni, and O atoms, respectively.

The multidimension appearances of the nine NiO_2_/Ni*^n^*/Pd nanocatalyst models proposed in this work are summarized in **Figure** **1**, these proposed designs refer to and match our prior NiO_x_‐Pd nanocatalyst experiments^[^ ^57^, ^62^ ^]^ (Detailed descriptions and explanations are provided in Note S1, Supporting Information). For brevity, the NiO_2_/Ni*^n^*/Pd systems are simplistically represented as the “N_#_” in the follow‐up discussions, that is, Ni_1_, Ni_2_, Ni_3_, Ni_4_, Ni_7_, Ni_10_, Ni_13_, Ni_1ML_, and Ni_2ML_ corresponding to Figure 1b–j.

The calculated system formation energy (*E*_f_) is used to assess the structural stability and rationality of the NiO_2_/Ni*^n^*/Pd surface models. Figure S1 (Supporting Information) provides all the associated *E*_f_ of the proposed model structures. The nine NiO_2_/Ni*^n^*/Pd structures have extremely comparable negative *E*_f_ values that are in the range from ≈−0.37 to −0.40 eV, which is significantly lower than 0.19 eV of pure Pd(111) benchmark. This suggests that all of the proposed NiO_2_/Ni*^n^*/Pd nanocatalyst structures are energetically favorable and well‐structured, indicating the intruding Ni atoms can freely spread from the basal NiO_2_ into the Pd thin layers, which is also consistent with our previous experimental results.^[^ ^57^, ^63^ ^]^ Besides, the “single‐atom Ni” doping configuration features the lowest *E*_f_ of −0.402 eV among all the configurations of interest, which means the Ni_1_ model is probably the experimentally most attainable structure of the raised designs.

### 3.2 Adsorbability of Atomic Oxygen and Hydroxyl Radical

On the grounds of the Sabatier principle concerning the ORR,^[^ ^25^ ^]^ the bonding strength (quantified by *E*_ads_) between an ideal catalyst and its adsorbed O* (the most critical ORR intermediate) ought to be at a befitting magnitude, i.e., sufficiently strong to easily obtain intermediates O* while keeping comparatively weak to let those O* break loose efficiently. Therefore, we used the calculated adsorption energy of O* (*E*_ads_‐O*), which is regarded a pivotal characteristic for forecasting the ORR activity of multifarious transition metals catalysts,^[^ ^24^, ^64^, ^65^, ^66^ ^]^ to initially assess the ORR patterns of the nine N_#_ model catalyst. Hereon, a calculated minus *E*_ads_ implies a spontaneous binding, i.e., a thermodynamically favorable adsorption behavior, and vice versa (details about the atomic adsorption configurations and descriptions about the site‐labels and corresponding landing locations for the O* and OH adsorption sites are supplied in Figure S2 and Note S2, Supporting Information).

**Figure 2:**
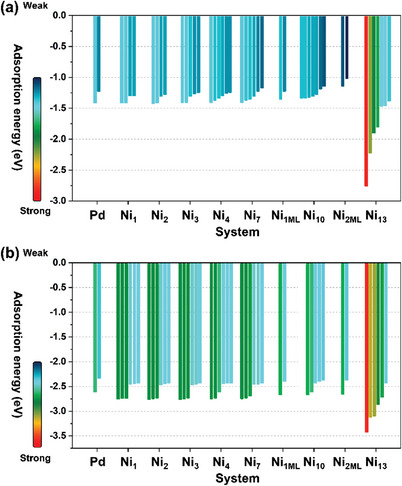
a,b) Adsorption‐energy diagrams of absorbed atomic O (*E*_ads_‐O*) and OH radical (*E*_ads_‐OH) at various threefold hollow sites of different models.

From **Figure** **2**a, the Pd(111) benchmark owns the *E*_ads_‐O* in the range of −1.22 to −1.41 eV, where its Pd‐hcp sites with more moderate *E*_ads_‐O* (−1.22 eV) can be regarded as the more preferable sites for ORR. Whereas, the *E*_ads_‐O* of the eight N_#_ systems (except Ni_13_) roughly show a tendency of gradually decreasing from Ni_1_ to Ni_2ML_, i.e., for their respective M‐h sites, the trend of the O* bonding strength generally follows the sequence: Ni_1_ (−1.29 to −1.30 eV) > Ni_2_ (−1.28 to −1.29 eV) > Ni_3_ (−1.25 to −1.30 eV) > Ni_4_ (−1.24 to −1.30 eV) > Ni_7_ (−1.18 to −1.30 eV) > Ni_1ML_ (≈−1.21) > Ni_10_ (−1.14 to −1.27 eV) > Ni_2ML_ (≈−1.02 eV). Compared to shell‐component Pd, the *E*_ads_‐O* observed on these eight N_#_ systems evidently have a wider catalytic selectivity scope, and concurrently exhibit a trend of stepwise diminishing with the bottom two Pd layers (i.e., second and third Pd layer) completely replaced by Ni atoms/layers (i.e., the Ni_2ML_). More explicitly, the *E*_ads_‐O* at the proposed optimal hcp sites of the Ni_1_ to Ni_4_ (−1.24 to −1.30 eV) perform slightly stronger adsorbability than that of the benchmark Pd(111) (−1.22 eV), which predicts a more easily O_2_ dissociation with lower activation barriers to break the tough O—O bond compared to Pd.

In contrast, the rest of the Ni_7_ to Ni_2ML_ show the relatively weaker *E*_ads_‐O* (−1.02 to −1.18 eV) than that of the Pd(111), which assumes a harder O_2_ splitting but a potential easier O* hydrogenation than the others. It is worth noting that the Ni_13_ significantly features the strongest *E*_ads_‐O* with the largest range from −1.39 to −2.77 eV, which is mainly attributed to the distinguishing arrangement of its doped thirteen Ni atoms, where its upper three Ni atoms incorporated into the outermost Pd layer exposing outside straightforwardly leading to the undesired metal Ni's adsorbability with severely excessive *E*_ads_‐O* nearly −3 eV, rather than to improve the *E*_ads_‐O* of metal Pd via the synergistic effect between the overlying Pd and the underneath Ni just like the other Ni_#_ systems (corresponding values of the *E*_ads_‐O* at various adsorption sites of each surface model are presented in Table S1, Supporting Information).

Analogically, from Figure 2b, compared to the adsorption energy of adsorbed OH radical (i.e., *E*_ads_‐OH) of the referential Pd(111) (−2.35 to −2.62 eV), the eight proposed N_#_ systems (# = 1–4, 7, 1ML, 10, 2ML, and 13) are observed to reveal slightly stronger *E*_ads_‐OH (−2.37 to −2.78 eV), which is likely to favor the O* hydrogenation reaction due to the strong OH binding is generally conducive to H—O—H bond breaking of H_2_O to obtain more free H* for O* reduction^[^ ^67^ ^]^), but not impede the OH* desorption (as a double‐edged sword, excessively strong OH binding can hinder the OH* desorption that causing the catalyst poisoning). As for the Ni_13_, it features a similar trend to its *E*_ads_‐O* distribution with a significantly wider *E*_ads_‐OH range and stronger OH bond strength due to the presence of three exposed Ni atoms on its outermost surface (detailed data of *E*_ads_‐OH see Table S1, Supporting Information). Besides, it can be observed that the *E*_ads_‐OH varies essentially not as rather sensitively as the *E*_ads_‐O* does with the doped Ni atoms changing, which also corroborates the key descriptive role of the *E*_ads_‐O* in the ORR characterization outweighs the OH or other ORR intermediates.

### 3.3 *d*‐band Model, Density of States, and Charge Migration

**Figure 3:**
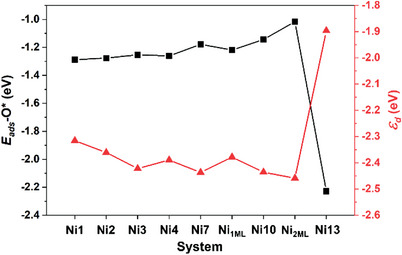
Calculated adsorption energies of atomic oxygen (*E*_ads_‐O*, eV) as a function of the *d*‐band centers (*ε*_d_, eV) at the involved surface atoms (M‐hcp sites) of the proposed model catalysts.

The volcano‐shaped dependence from the *d*‐band model theory^[^ ^22^, ^37^, ^68^, ^69^, ^70^ ^]^ on the *d*‐band center (*ε*_d_) offset of surface atoms versus their *E*_ads_‐O* has gained broad acceptance in transition‐metal catalysis circles regarding the ORR activity prediction/understanding, where the *ε*_d_ value is a very useful character to assess the coupling degree between the *d* electrons of a transition metal surface and valance electrons of its adsorbed atomic oxygen (O*) through the calculated *E*_ads_‐O*. Specifically, when the surface metallic atoms are compressed/stretched, the overlap of *d* orbitals between neighboring metal atoms increases/decreases, causing the broadened/narrowed bandwidth and the downshift/upshift of the *ε*_d_ versus the Fermi level (*E*_Fermi_) to keep the *d*‐band filling, thus leading to the more/less occupied states of *d* orbitals and the resulting weaker/stronger chemisorption for the intermediate O*. **Figure** **3** depicts the calculated correspondence relationship between the calculated *ε*_d_ of the selected three‐fold hcp site and the value of its *E*_ads_‐O*. For comparison, the selected triatomic hcp site for all models correspond to the same central locus on outermost surfaces (details about the selected M‐hcp sites and corresponding values of the *ε*_d_ and *E*_ads_‐O* are listed in Tables S2 and S1, Supporting Information). From Ni_1_ to Ni_2ML_, the *E*_ads_‐O* (the black line) varies upward (weaken) progressively from −1.30 to −1.02 eV, and then abruptly goes down (strengthen) to Ni_13_ (−2.23 eV) due to its hcp site replaced by Ni atoms. On the other side, the *ε*_d_ values fluctuate in a tendency completely reverse to the *E*_ads_‐O*, i.e., gradually drift downward from −2.32 to −2.46 eV (from Ni_1_ to Ni_2ML_), and then rise steeply to −1.90 eV of Ni_13_. Hence, there is an almost exactly opposite correspondence between the calculated *E*_ads_‐O* and *ε*_d_, which also validates well the deduction mentioned above that the farther the locus of *ε*_d_ is from *E*_Fermi_, the weaker the obtained *E*_ads_‐O*. As for the pure Pd(111) benchmark, its calculated *ε*_d_ is around −1.77 eV, which is in accordance with the literature as well as our previous works (see in Table S2, Supporting Information).^[^ ^71^, ^72^, ^73^ ^]^ Therefore, based on the *d*‐band model corollary, the calculated *ε*_d_ of the Ni_#_ systems (−1.90 to −2.46 eV) at the involved hcp sites lie below the benchmark Pd (−1.77 eV), which suggests that our proposed N_#_ catalysts are highly anticipated to outweigh the noble metal Pd(111) in terms of the ORR activity.

**Figure 4:**
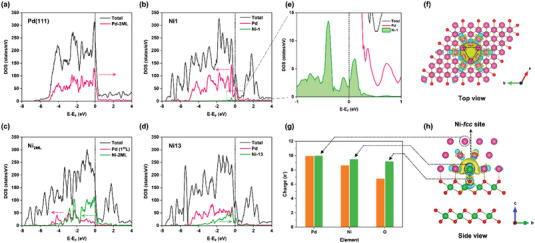
Projected density of states (PDOS) for the surface Pd layer and the Ni atoms intercalated in the Pd layer of a) Pd(111), b) Ni_1_, c) Ni_2ML_, and d) Ni_13_ surface models; for clarity, the total DOS (TDOS) is projected onto all orbitals of total atoms that constitute the model catalyst, while the PDOS is projected onto the *d* orbitals of the involved atoms. e) A magnified image taken from (b). Charge density difference of the single‐atom‐Ni doped NiO_2_/Pd system from f) the top view and h) the side view, where the yellow and blue areas, respectively, mean the charge increase and depletion. g) Bader charge (e^−^) of the doped Ni atom, the Pd atom in the surface triatomic (Ni‐fcc) site right above it, the O atom coordinates with it below (the three green bars), while the e^−^ of a Ni atom in the NiO_2_, a Pd atom in the pure Pd, a O atom in the NiO_2_ correspond to the three orange green bars.

To better understand the physical mechanism behind this dependency between *ε*_d_ and *E*_ads_‐O*, the density of state (DOS) as well as the charge migration calculations are conducted to evaluate how the synergistic effect of the NiO_2_‐Pd structure and the interfacial Ni*^n^*‐doping influence the surface electronic structures of the proposed NiO_2_/Ni*^n^*/Pd systems. **Figure** **4**a shows the total and projected DOS of the pure Pd(111) model, which is well consistent with the literature.^[^ ^74^, ^75^, ^76^ ^]^ The projected DOS of the Ni_1_ model in Figure 4b exhibits that as the main states contributor around the Fermi level, the Pd *4d* orbitals of Ni_1_’s three Pd layers (the purple line) have a significant shift toward lower energy levels compared to the corresponding Pd orbitals of benchmark Pd(111), which indicates that more occupied state electrons are obtained to broaden its surface integral *d*‐bandwidth and to sink the corresponding *ε*_d_. Meanwhile, the doped Ni^SA^ is also observed to contribute its major Ni *3d* orbitals (the green line) to its occupied states, which is favorable to the *ε*_d_ migration to the deep levels. Figure 4e highlights the overwhelming majority of Ni^SA^ *3d* states in Ni_1_ accompanied with the strongest orbital peak are located just below the *E*_Fermi_ (−1 to 0 eV), which confirms important role of Ni^SA^ in Ni_1_ for facilitating surface electron exchange and *d*‐band sinking. As the number of doped Ni atoms increases to two monolayers’ substitution (i.e., Ni_2ML_) in Figure 4c, the major contribution to the occupied states is strongly hybridized Ni *3d* and Pd *4d* orbitals, where both of the two orbitals feature a tendency to shift toward lower levels compared to Ni_1_ and Pd(111), which results in the lowest *ε*_d_ position (see Figure 3). As for Ni_13_ from Figure 4d, its Pd *4d* orbitals still feature an analogical distribution to that of the Ni_1_. However, its Ni *3d* orbitals exhibit a distinct trend to move towards higher levels with a narrower bandwidth due to its exclusive exposed Ni atoms on outermost Pd surface, thereby causing a *ε*_d_ elevation (corresponding projected DOS results for all proposed NiO_2_/Ni*^n^*/Pd model catalysts are provided in Figure S5, Supporting Information). As a result, the calculated DOS results trace out the significant synergistic effect triggered by the NiO_2_/Pd heterostructure and the interfacial Ni heteroatomic doping on the regulation of the surface electronic structures of the proposed NiO_2_/Ni*^n^*/Pd systems, which not only reveal the underlying factors for the *ε*_*d*_ shift, but also solidates our theoretical prediction for the adsorption properties and ORR activity.

Furthermore, the underlying charge relocation between the interfacial intercalation of Ni^SA^ and its surrounding atoms in the Ni_1_ system was evaluated by charge density difference and Bader charge calculations on behalf of the NiO_2_/Ni*^n^*/Pd series. From Figure 4f,h, the 3D plots of charge density difference exhibit a significant tendency of charge agglomeration over the doped Ni^SA^. More explicitly, extra available charge injected to the Ni‐*fcc* site is believed can enhance the capture capacity of the electroneutral O_2_ and H_2_O to promote their chemisorption, simultaneously, introduce the repulsive force for the electronegative O* and OH to facilitate their desorption, therefore, synergistically boost the two ORR steps moving forward in the positive direction. Bader charge population was then calculated to quantify the charge transfer of the specific atoms/elements, as shown in Figure 4g,h, which demonstrates that the additional available charge obtained to the Ni^SA^ and its above Pd atoms due to the synergistic effect (strain, ligand, and geometric effects) significantly enables the surface Ni‐*fcc* site a superior redox activity for Ni_1_ system compared to pure Pd. Meanwhile, a large increase in the excess charge of the O atom coordinated with the Ni^SA^ means a much stronger Ni—O electrovalent bond, i.e., a thermodynamically more stable core–shell doping structure. (More detailed discussions about the charge transfer/relocation scenarios (around Ni^SA^) and their impact on the ORR activity of Ni_1_ systems are provided in Note S3, Supporting Information.)

### 3.4 ORR Mechanisms on the NiO_2_/Ni*^n^*/Pd Systems

**Figure 5:**
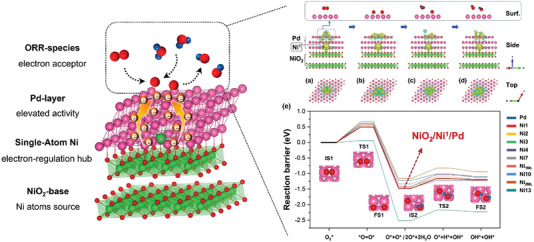
Left: schematic diagram of the charge transfer process between NiO_2_‐base, interfacially intercalated single‐atom (SA) Ni, outward Pd‐shell atoms, and surface ORR species of the NiO_2_/Ni^1^/Pd system. Lower right: free energy diagram of complete ORR paths in alkaline medium on the proposed model surfaces, including the respective key O_2_ dissociation structures (IS1, TS1, and FS1) and O* hydrogenation structures (IS2, TS2, and FS2). Upper right: schematics of the electron density difference between the doped single‐atom Ni, Pd‐shell atoms and surface adsorbates of the representative NiO_2_/Ni^1^/Pd model: (a) to (b) corresponding to the IS1 to FS1, and (c) to (d) corresponding to the IS2 to FS2, where the yellow and blue areas represent the charge density increase and decrease, respectively. e) Free energy diagram of complete ORR paths on the proposed model catalysts in this work.

To ascertain the ORR mechanism of our proposed NiO_2_/Ni*^n^*/Pd systems under base conditions, the simulated kinetic CI‐NEB approach is used to seek the reaction activation barriers of the respective O_2_ dissociation substep (i.e., ∆*E*1) and O* hydrogenation substep (i.e., ∆*E*2), which can directly characterize the catalytic dynamic behaviors for the cathode catalysts. The results are also compared with the benchmarking Pd(111) (Details about the atomic adsorption structure and descriptions for the stable IS and FS of the two substeps on the proposed Ni_#_ surface models are provided in Figures S3 and S4 and Note S4, Supporting Information, respectively). The calculated reaction coordinates of the two ORR subroutes and the corresponding values of ∆*E*1 and ∆*E*2 corresponding to the TS of each reaction route are presented in the lower right of **Figure** **5**, Table S3 and Figure S6 (Supporting Information), respectively. First, for the rate‐determining step (RDS), the O_2_ dissociation path on each catalyst is thermodynamically spontaneous (exothermic) judged by the energy relation between IS1 and FS1. Ni_1_ reveals the lowest ∆*E*1 of only 0.49 eV among all (0.49–0.67 eV) except for Ni_13_. In comparison with the benchmark Pd(111)’s 0.62 eV, the proposed Ni_1_ with minimal Ni doping (Ni^SA^) reduces the O—O bond cleavage obstacle by 0.13 eV (over 20% decline), which contributes to the improved efficiency of initial O_2_ splitting and the intermediates O* acquisition of the catalyst, but less noble Pd usage. As the number of doped Ni atoms increases (Ni_2_ to Ni_2ML_), their computed ∆*E*1 for O_2_ splitting are observed to augment progressively from 0.50 to 0.67 eV, but only Ni_2ML_ with maximal Ni doping shows ∆*E*1 (0.67 eV) softly exceeding that of pure Pd (0.62 eV) by less than 0.05 eV due to its weakest *E*_ads_‐O*. In addition, Ni_13_ is observed to have a sharply dropped ∆*E*1 to about 0.1 eV compared with the others (0.49–0.67 eV), which is evidently attributed to its considerably enhanced O* bonding strength (*E*_ads_‐O*, −1.39 to −2.77 eV) that almost erases its O—O breakage barriers. Subsequent is the “O* hydrogenation,” where the obtained O* will be reduced by H_2_O into absorbed OH. Contrary to the preceding O_2_ splitting, O* hydrogenation for all proposed Ni_#_ systems and pure Pd are thermodynamically nonspontaneous reactions (endothermic). As the benchmark one in the alkaline, Pd(111) features a low ∆*E*2 of 0.21 eV (corresponding to the TS2). By comparison, Ni_1_ shows a slightly higher ∆*E*2 (0.30 eV) than that of pure Pd due to its moderately stronger *E*_ads_‐O*, which results in a little harder than Pd but still quite easy O* desorption. The same holds true for the rest Ni_#_ systems that own a relatively low ∆*E*2 range of 0.24–0.34 eV. Another point of concern is Ni_13_, its dramatically increased *E*_ads_‐OH balances the abnormal increase of ∆*E*2 (to just 0.40 eV) caused by its excessively increased *E*_ads_‐O*, yet, the too strong OH binding may impede OH* desorption away from surface sites. This unusual ORR kinetic behavior observed on the Ni_13_ still needs further investigation. These results verified that our proposed NiO_2_/Ni*^n^*/Pd systems with low Pd loading can exhibit superb ORR activity outperforming the benchmarking Pd(111) via interfacially doping Ni. Specifically, the NiO_2_/Ni^1^/Pd with Ni_SA_ intercalating in overlying Pd layer performs the optimal kinetic behavior in the two key ORR substeps (i.e., ∆*E*1, 0.49 eV and ∆*E*2, 0.30 eV).

Moreover, for ease of understanding the improvement mechanism in ORR, the charge transfer scenarios behind the two kinetic stages between the doped Ni^SA^, surface atoms and the relevant absorbates for the representative optimal Ni_1_ system were investigated, as shown in the upper right of Figure 5. During the four key stages IS1 (O_2_*), IS2 (O* + O*), FS1 (H_2_O* + O*), and FS2 (HO* + HO*), though buried at the bottom of the three‐layer Pd shell, the Ni^SA^ still has a significant charge interaction with the surface adsorbates through the Pd atoms sandwiched in the middle. In more vivid words, the interfacially intercalated Ni element seems to act more like an “electron‐regulation hub” in the NiO_2_/Ni^1^/Pd system, whose catalytic activity is mainly featured by its outward Pd shell and a Ni^SA^ interior, eventually achieve: 1) activity improvement of outermost layer Pd atoms within a local domain via the electronic structure adjustment, 2) cover‐up the disadvantage of less activity and easier passivation of cheap Ni compared to Pd, and 3) stability enhancement of the core‐shell‐type NiO_2_/Pd structure via stronger ionic bonding between interfacially doped Ni^SA^ and core‐part O atom. Since the atoms in subsurface or deeper layers of catalysts usually play an inferior role in the ORR, the partial replacement of expensive Pd in subsurface‐layer(s) with much cheaper Ni is believed can not only greatly reduce the cost of nanocatalysts, more notably, the local synergistic effect introduced by the interior Ni^SA^ triggers a prominent reactivity and selectivity on catalyst surface, which realize the bifunctionality of the ORR improvement as well as the cost control. The schematic of charge transfer process inside the optimized NiO_2_/Ni^1^/Pd system and the chemical interactions between the catalyst and ORR species is illustrated in the left of Figure 5.

### 3.5 Correspondence between Adsorption Energies and Reaction Barriers for the NiO_2_/Ni*^n^*/Pd Systems

**Figure 6:**
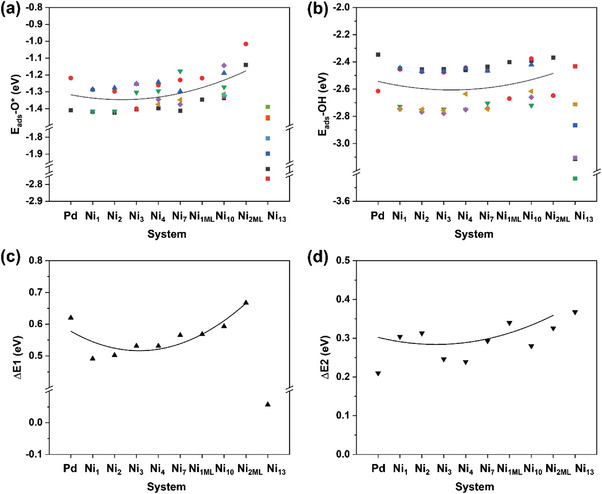
Variation trends of the a) *E*_ads_‐O*, b) *E*_ads_‐O*, c) Δ*E*1, and d) Δ*E*2 for all proposed model catalysts.

Based on the above results involving the adsorption energies of O* (*E*_ads_‐O*) and OH (*E*_ads_‐OH), the ORR activation barriers of the O* dissociation (Δ*E*1) and O* hydrogenation (Δ*E*2) reactions, we found an underlying relationship between these four key factors and the corresponding model configurations. In a word, the very analogical fitting crescent‐shaped trendline for the distributions of *E*_ads_‐O*, *E*_ads_‐OH, Δ*E*1, and Δ*E*2 are observed from the pure Pd to Ni_2ML_, as shown in **Figure** **6**a–d, respectively, which well proves our predicted dependency as well as the reciprocal action between adsorption energy versus reaction barrier for the proposed Ni_#_ systems mentioned above (Detailed descriptions and discussions involving the variation relations between calculated *E*_ads_, Δ*E* and proposed structures are supplied in Note S5, Supporting Information). As a result, the calculated variations of the *E*_ads_‐O* and *E*_ads_‐OH, together with their respective Δ*E*1 and Δ*E*2 revealed a structure‒*E*_ads_‒Δ*E* correspondence between the adsorption strength (O* and OH), two‐step's barriers, and the corresponding NiO_2_/Ni*^n^*/Pd structures with the gradual infiltration of Ni atom(s) from the NiO_2_/Pd interface to the subsurface. In brief, the ORR activity basically continuously improves with the decreasing number of doped Ni atoms, where the optimal ORR activity is achieved at the doping level being minimized to the single‐atom scale. This conclusion is considered can be applied as a guideline in future theoretical and experimental research.

Comprehensively, concerning the proposed core/shell structured NiO_2_/Ni*^n^*/Pd with surface geometry identical to Pd(111) (i.e., Ni_1_ to Mi_2ML_), the gradual infiltration of Ni atom(s) from the NiO_2_/Pd interface (i.e., third Pd layer) to the subsurface (i.e., second Pd layer) is believed can 1) lead to the weakening of the *E*_ads_‐O*, which synchronously increase the Δ*E*1 of the first O_2_ dissociation step, 2) the weakening of the *E*_ads_‐OH also cause the slighted increased Δ*E*2 of the second O* hydrogenation step, 3) the weakened *E*_ads_‐O* can help to alleviate the Δ*E*2 increase for the second ORR stage, therefore, the obtained Δ*E*2 of the proposed Ni_#_ systems are observed to fluctuate within a very small range (≈0.1 eV), 4) the Ni_13_ exhibiting an abnormal ORR behavior is assumed to perform superb ORR activity due to its naked Ni on outermost surface, but still needs further investigation and demonstration, and 5) the Ni_1_ system with Ni^SA^ interfacially intercalated between Pd‐shell and NiO_2_‐core features the optimized ORR activity compared to the rest, let alone pure Pd benchmark.

### 3.6 Doping‐Depth Effect of Single‐Atom Ni in the Pd Layer on the ORR

Based on the foregoing, we have proved that the Ni_1_ with only single‐atom Ni doping in its Pd‐shell featuring the optimum ORR performance via cross‐referencing the result of the surface adsorption properties, electronic structures, charge relocation, and the ORR kinetics behavior. However, theoretical and experimental studies had also indicated that the electronic influence and strain effect of the substrate on the topmost surface of transition‐metal catalysts would begin to vanish gradually for the coverages of *n* > 3 ML.^[^ ^44^, ^77^, ^78^ ^]^ Therefore, to screen the optimal intercalation scheme of Ni^SA^ and to achieve a deepgoing understanding regarding the architectural feature and catalytic property of the optimal NiO_2_/Ni^1^/Pd system in atomic level, herein, the doping depth effect of Ni^SA^ in Pd layer of the NiO_2_/Pd structure on ORR is further explored targetedly.

**Figure 7:**
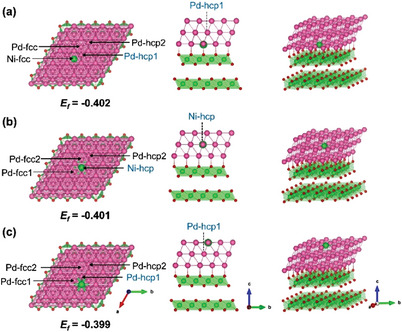
Top, side, and oblique views for the three types of NiO_2_/Ni^1^/Pd model with the single‐atom Ni doping at different depths of Pd layers: a) Ni^SA^‐3^rd^, b) Ni^SA^‐2^nd^, and c) Ni^SA^‐1^st^. For clarity, the key threefold M‐hcp sites for O* of each surface model are marked in bold blue, together with the calculated formation energy (*E*_f_) of each slab.

**Figure 8:**
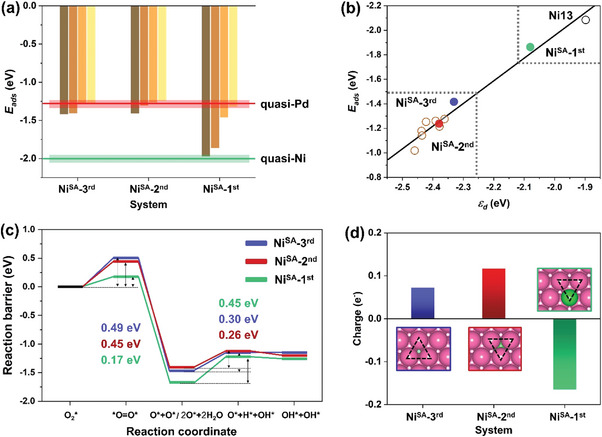
Calculated various physical‐chemical properties and adsorption energies of the three Ni_1_ models. a) *E*_ads_‐O* at assigned threefold sites of the three models. b) Calculated *E*_ads_‐O* a function of the *d*‐band centers (*ε*_d_) at the involved surface atoms (M‐hcp sites) of the three catalysts. c) Free energy diagram of complete ORR paths on the proposed three model surfaces. d) Excess charge (e^−^) of the assigned surface triatomic site (M‐hcp) of the proposed three models.

**Figure** **7**a–c illustrated the proposed three NiO_2_/Ni^1^/Pd systems corresponding to a Ni^SA^ intermixed in the third Pd layer, second Pd layer, and first Pd layer in the NiO_2_/Pd core/shell configuration, which are denoted as the “Ni^SA^‐3^rd^”, “Ni^SA^‐2^nd^”, and “Ni^SA^‐1^st^”, respectively. The calculated *E*_f_ of the three model slabs listed in Figure 7 show that the three proposed Ni_1_ systems (Pd‐Ni^SA^ solid‐solution structures) are energetically reasonable due to their almost identical *E*_f_ values within less than 0.01 eV/atom. Meanwhile, the Ni^SA^ is thermodynamically slightly easier to replace the Pd atom in the or second or third Pd layer compared to in outermost Pd layer (first), which is also consistent with our previous experimental observations.^[^ ^57^, ^63^ ^]^ **Figure** **8**a shows a very similar variation range of *E*_ads_‐O* on the Ni_SA_‐3^rd^ and Ni_SA_‐2^nd^ systems due to their identical outermost‐layer appearance to Pd(111). Nevertheless, the Ni^SA^ doping depth still leads to a slight difference on the *E*_ads_‐O*, specifically, the second‐layer doping of Ni^SA^ has a more significant impact on the electronic structure of the topmost Pd layer compared to the third‐layer doping. Specifically, the doped‐Ni atom in the second Pd layer with smaller Wigner radius can introduce a salient compressed lattice zone into the outermost Pd surface above it, which triggers a stronger *ε*_d_ downshift of the involving surface Pd atoms and the attenuation of the corresponding *E*_ads_‐O* (i.e., the calculated average Pd—Pd bond length of the surface triatomic Pd‐hcp site above the Ni^SA^ of the Ni_SA_‐2^nd^ (2.75 Å) is ≈0.04 Å shorter than that of the Ni_SA_‐3^rd^ (2.79 Å), which means the Pd‐hcp site of Ni_SA_‐2^nd^ is subjected to 14% more compressive strain than that of the Ni_SA_‐3^rd^, as shown in Figure S7, Supporting Information). For another, the Ni^SA^‐1^st^ shows a remarkably increased *E*_ads_‐O* from −1.33 to −1.97 eV mainly due to its doped Ni^SA^ exposed on the topmost Pd layer that is similar to the aforementioned Ni_13_ (detailed *E*_ads_‐O* values of the three Ni_1_ systems see Table S4, Supporting Information). Qualitatively, the Ni^SA^‐2^nd^ and Ni^SA^‐3^rd^ further improve the surface O* binding strength for the robust Pd (i.e., quasi‐Pd level), while the Ni^SA^‐1^st^ exhibit an *E*_ads_‐O* range within a large fluctuation between metal Pd and Ni. Figure 8b also verifies the linear relation between the *ε*_d_ at the involved surface atoms and their corresponding *E*_ads_‐O* (the selected hollow sites are marked in Figures 3 and 7). It indicates that the NiO_2_/Ni*^n^*/Pd systems with the identical surface appearance (including Ni^SA^‐2^nd^ and Ni^SA^‐3^rd^) tend to land in the superior coordinate range (left bottom) appropriate for the ORR than those of the Ni^SA^‐1^st^ and Ni_13_ systems with the Pd/Ni hybrid surfaces, according to the general trend of the classical *ε*_d_—*E*_ads_ correlation on the ORR.^[^ ^37^ ^]^ Meanwhile, the Pd‐hcp site of Ni^SA^‐2^nd^ features a catalytically more preferable coordinate to Ni^SA^‐3^rd^, which is due primarily to the more significant synergistic effect (electronic and strain) on the surface triggered by the doped Ni^SA^ in the second Pd layer than that in the lower third Pd layer. Thus, the ORR activity trend described from our calculated *ε*_*d*_–*E*_ads_ correlation and the *E*_ads_‐O* plot basically follows the sequence: Ni^SA^‐2^nd^ > Ni^SA^‐3^rd^ > Ni^SA^‐1^st^.

Simulated ORR reaction coordinates of the two ORR subroutes with the corresponding energy barrier (TS/∆*E*) for the three “Ni_1_” are presented in Figure 8c. Ni^SA^‐2^nd^ catalyst reveals a quite low ∆*E*1 in the RDS “O—O dissociation” (0.45 eV), which is even 0.04 eV smaller than that of Ni^SA^‐3^rd^ (0.49 eV). Not only that, the ∆*E*2 in the subsequent “O* hydrogenation” of Ni^SA^‐2^nd^ is also the smallest (0.26 eV) among the three, which remains lower than that of Ni^SA^‐3^rd^ (0.30 eV) by 0.04 eV. Besides, although the Ni^SA^‐1^st^ shows a fairly small ∆*E*1 of 0.17 eV compared with the other two, its dramatically reduced activation barrier of O* adsorption is mainly derived from the stronger oxidation capacity of its Ni^SA^ embedded in the outermost surface, which undesirably leads to an elevated obstacle in the following O* desorption and affects the ORR efficiency like aforementioned Ni_13_. Therefore, the transition‐state calculations kinetically verify the Ni^SA^ doping in the Pd subsurface contributing the most to catalytic synergy on the improvement of both O_2_ dissociation and O* desorption stages. As a comparison with another benchmark platinum catalyst in alkaline FCs, the key RDS barriers of the NiO_2_/Ni*^n^*/Pd systems (∆*E*1, 0.50–0.67 eV) are quite lower than that of the precious Pt(111) benchmark (1.04 eV),^[^ ^56^, ^79^ ^]^ where the ∆*E*1 further descends dramatically by 84% to 53% since the Ni atom(s) are intercalated as a single atom on the Pd layer (∆*E*1, 0.17–0.49 eV from Ni^SA^‐1^st^ to Ni^SA^‐3^rd^, as shown in Figure S8, Supporting Information).

Besides, from a thermodynamic point of view in Figure S9a (Supporting Information), the calculated potential energy surface (∆*E* ^T^ = IS − FS) of O_2_ dissociation (∆*E* ^T^1) and hydrogenation (∆*E* ^T^2) thermodynamically shows that the first O_2_ dissociation reactions for all three Ni1 models are thermodynamically spontaneous (i.e., ∆*E* ^T^1 > 0, 1.42–1.67 eV). On the contrary, the second O* hydrogenation reactions are all thermodynamically nonspontaneous processes (i.e., ∆*E* ^T^2 < 0, −0.21 to −0.42 eV), which means that there is a far greater risk of a reverse O* hydrogenation for the three catalysts compared to their preceding O_2_ dissociation. Notably, Ni^SA^‐2^nd^ features the ∆*E* ^T^2 (−0.21 eV) closest to zero along the positive direction of the second step, indicating that the O* hydrogenation on Ni^SA^‐2^nd^ is thermodynamically more stable to proceed among the three models, especially much more positive than −0.42 eV of Ni^SA^‐1^st^ catalyst. From another kinetic point of view in Figure S9b (Supporting Information), the calculated reverse reaction barriers (∆*E* ^R^ = TS − FS) of O_2_ dissociation (∆*E* ^R^1) and hydrogenation (∆*E* ^R^2) kinetically quantify the difficulty of unwanted backward reactions of the two ORR steps. The ∆*E* ^R^1 of the three catalysts (1.85–1.86 eV) is much higher than their corresponding forward ∆*E*1 (0.17–0.49 eV), indicating that the forward process of O_2_ dissociation is kinetically pretty stable. While for the subsequent O* hydrogenation, the ∆*E* ^R^2 of all three Ni1 catalysts (0.03–0.05 eV) shows a similar but slightly small magnitude to the corresponding forward ∆*E*2 (0.26–0.45 eV), which suggests the possible risk of reverse OH* de‐hydrogenation causing the intermediate O* blocking. In vivid words, O* hydrogenation is more like a free two‐way highway. Likewise, Ni^SA^‐2^nd^ is observed to have the highest ∆*E* ^R^2 (0.05 eV) than Ni^SA^‐1^st^ (0.03 eV) and Ni^SA^‐3^rd^ (0.04 eV), meaning that the O* hydrogenation on Ni^SA^‐2^nd^ is kinetically more difficult to proceed backward than the other two. As a result, the Ni^SA^‐2^nd^ system is believed to feature a more reasonable and efficient ORR behavior from both thermodynamic and kinetic perspectives, especially in avoiding the blockage of intermediates O* between the two ORR substeps (caused by reverse reaction) and the desorption of final products OH* (caused by the strong *E*_ads_‐O* and *E*_ads_‐OH), which should be the severe issues faced by the Ni^SA^‐1^st^ system.

Bader charge analysis in Figure 8d deeply uncovers that the excess charge (*n*e^−^) obtained from the surface triatomic sites right above the doped Ni^SA^ of the three systems is roughly in this sequence: Ni^SA^‐2^nd^ (0.118 e^−^) > Ni^SA^‐3^rd^ (0.074 e^−^) > Ni^SA^‐1^st^ (−0.166 e^−^), where the *hcp*‐site of Ni^SA^‐2^nd^ features the most available (excess) charge among the three that proving its potential highest activity in the ORR. For another, the *hcp*‐site of Ni^SA^‐1^st^ exhibits a negative value of the excess charge (−0.166 e^−^) is due to the exposure of smaller electronegative Ni^SA^ (*χ* = 1.91) in the *hcp*‐site, resulting in the electrons extraction to its surrounding higher electronegative Pd atoms (*χ* = 2.20). Since the charge‐deficient *hcp‐*site would tend to extract electrons from O atoms to compensate for its *d* electrons loss, which in turn causes the key intermediates O* the metastable state due to the valence charges balance of octet rule, eventually giving rise to the prone surface‐site passivation and inadequate ORR efficiency.

By cross‐referencing the energetics adsorption properties, the corollaries of *d*‐band model, kinetics simulation verification, and the in‐depth charge transfer scenario, we intrinsically elucidate the influence of Ni^SA^‐doping depth in the Pd shell (from first to third layer) on the ORR performance, that is, the Ni^SA^ intercalated in the subsurface (Ni^SA^‐2^nd^) is believed to trigger the most fruitful Ni^SA^/Pd^ML^ synergy combining the electronic influence and strain effect that significantly alter the surface electronic structure and synchronously optimize the redox properties of the NiO_2_/Pd structure, followed by the Ni atom infiltrating into the third layer of Pd‐shell (Ni^SA^‐3^rd^), where the introduced synergistic effect between Ni^SA^ and Pd atoms begins to whittle for more than one Pd layer coverage. While, the Ni^SA^ incorporated into the outermost layer (Ni^SA^‐1^st^) exhibits an abnormal ORR behavior, which is considered unfavorable for the enhancement of ORR performance compared to the other two. Overall, the potential ORR activity of three NiO_2_/Ni^1^/Pd systems generally follows the sequence: Ni^SA^‐2^nd^ > Ni^SA^‐3^rd^ > Ni^SA^‐1^st^.

## 4 Conclusions

In this study, the non‐Pt nanocatalyst composed of NiO_2_ supported Pd surface configurations with various degrees of Ni atoms interfacially intercalated in the Pd layer, i.e., the NiO_2_/Ni*^n^*/Pd systems (where *n* denotes 1, 2, 3, 4, 7, 1ML, 10, 2ML, and 13), are constructed and their physical and chemical properties and the corresponding dimension and depth effects of doped‐Ni on the ORR performance are systematically cross‐assessed by using the DFT calculations.

Our results demonstrate that the crucial *E*_ads_‐O* index of the NiO_2_/Ni*^n^*/Pd systems shows an evident tendency of gradually decreasing from Ni_1_ to the Ni_2ML_ (−1.02 to −1.30 eV) as the bottom two Pd layers substituted by Ni atoms. Together, the slightly enhanced adsorption energy of OH radical (*E*_ads_‐OH) observed in Ni_1_ is forecasted to contribute to the O* hydrogenation compared to pure Pd. The subsequent *ε*_d_, projected DOS and physical charge transfer calculations respectively describe the well coincidence relation between the calculated *ε*_d_ of the threefold site and its *E*_ads_‐O* values as well as the state's contribution of the adjusted Pd layer and the doped‐Ni, which confirm the significant synergy on the surface electronic‐structure regulation for the NiO_2_/Ni*^n^*/Pd system, indicating a superior level of ORR performance to the costly benchmark Pd. TS calculations of the reaction pathway kinetically verify the above prediction of *E*_ads_‐O*/‐OH on the ORR activity. Especially, the optimized Ni_1_ system performs the optimum ORR behavior with the lowest Δ*E*1 (0.49 eV) and very moderate Δ*E*2 (0.30 eV) compared with benchmark Pd (0.62 and 0.21 eV). The charge transfer scenarios behind the two key ORR stages uncover the focal role of interfacially intercalated Ni^SA^ the “electron‐regulation hub” in the NiO_2_/Ni^1^/Pd system, which tunes the electronic structure of the Pd‐shell layers and concurrently increases the heterogeneous interface bonding via stronger ionic bonding. Through stepwise cross‐level demonstration between the outcomes of the *E*_ads_, *d*‐band model, projected DOS and reaction coordinates simulation, a noteworthy Structure‒*E*_ads_‒Δ*E* relationship is excavated for our proposed Ni_#_ systems, which reveals the important correspondence between the four key ORR factors and related catalyst structures. Furthermore, we explore the influence of the Ni^SA^ doping depth for the NiO_2_/Ni^1^/Pd system on ORR, which intrinsically elucidates that the Ni^SA^ incorporated into the subsurface of Pd (Ni^SA^‐2^nd^) raises the remarkable Ni^SA^/Pd^ML^ synergy combining the electronic influence and strain effect to improve the ORR activity, whereas this uplifting synergy begins to vanish for more than one Pd coverages (i.e., Ni^SA^‐3^rd^). Ni^SA^‐1^st^ performs an unusual low ∆*E*1 and a relatively higher ∆*E*1 due to its several Ni atom exposed on the catalyst surface, the same applies to the Ni_13_.

In conclusion, interfacial doping few inexpensive Ni in costly subsurface‐Pd‐layer in the NiO_2_/Ni^1^/Pd systems can motivate a unique local synergy accompanied by a remarkably improved reactivity and selectivity to the catalysts compared with benchmark Pd(111), particularly, the NiO_2_/Ni^1^/Pd model with Ni^SA^ intercalated in the subsurface has been proven to be optimal configuration design, which realizes the anticipated bifunctionality of activity enhancement and cost control. By this means, the use of precious platinum in generally conceived “good commercial electrocatalysts” can be sharply cut down to “non‐Pt level” but with a superior overall performance. The concepts and findings presented in this work provide a precision evaluation of the process window for optimizing the composition and structure of multimetallic catalysts in practical experiments regarding the novel Pt‐free, high‐performance, long‐lasting, and environmentally‐friendly techniques.

## Conflict of Interest

The authors declare no conflict of interest.

## Author Contributions

T.‐Y.C. and J.‐P.C. conceived the overall project. H.L.L. and J.‐P.C. proposed and refined the model design. H.L.L. carried out all the DFT calculations. H.L.L., J.‐P.C., and T.‐Y.C. performed the corresponding data analysis. H.L.L., J.‐P.C., and T.‐Y.C. interpreted the results and wrote the manuscript. H.‐Y.T.C. provided ideas and suggestions on the DOS calculations and analysis. S.D. and J.J.C. offered auxiliary ideas about the nanocatalysts from the aspect of their previous experimental observations concerning nanomaterials and heterogeneous doped structures. A.H., Y.W.W., and Q.D. assisted in further discussions and proofreading.

## Supporting information

Supporting Information

## Data Availability

The data that support the findings of this study are available from the corresponding author upon reasonable request.
