# Supplementary material for: Atomic Scaled Depth Correlation to the Oxygen Reduction Reaction Performance of Single Atom Ni Alloy to the NiO2 Supported Pd Nanocrystal
Source: Adv Sci (Weinh). 2023 Feb 8;10(11):2207109. doi: 10.1002/advs.202207109 (PMC10104651; doi:10.1002/advs.202207109)
Supplement: Supplementary file 1 — Supporting Information [file ADVS-10-2207109-s001.pdf]

Supplementary Information for

# Atomic scaled depth correlation to the Oxygen reduction reaction performance of single atom Ni alloy to the $NiO_2$ supported Pd nanocrystal

*Haolin Li,[a,b,c] Sheng Dai,[e] Yawei Wu,[c] Qi Dong,[f] Jianjun Chen,[a] Hsin-Yi Tiffany Chen,[b] Alice Hu,[c,g*] Jyh-Pin Chou[d*] and Tsan-Yao Chen[b,h,i*]*

[a] *School of Materials Science and Engineering, Zhejiang Sci-Tech University, Hangzhou, 310018, P.R. China*
[b] *Department of Engineering and System Science, National Tsing Hua University, Hsinchu, Taiwan*
[c] *Department of Mechanical Engineering, City University of Hong Kong, Hong Kong SAR, P.R. China*
[d] *Department of Physics, National Changhua University of Education, Changhua 50007, Taiwan*
[e] *School of Chemistry & Molecular Engineering, East China University of Science and Technology, Shanghai, P.R. China*
[f] *Department of Electrical Engineering, Tsinghua University, Beijing 100084, P. R. China*
[g] *Department of Materials Science and Engineering, City University of Hong Kong, Hong Kong SAR, China*
[h] *Hierarchical Green-Energy Materials (Hi-GEM) Research Centre, National Cheng Kung University, Tainan 70101, Taiwan*
[i] *Department of Materials Science and Engineering, National Taiwan University of Science and Technology, Taipei 10617, Taiwan*

*** Corresponding author**: (A. H) Email: alicehu@cityu.edu.hk; (J.-P. C) Email: jpchou@cc.ncue.edu.tw; (T.-Y. C) Email: chencaeser@gmail.com; tsanyao@mx.nthu.edu.tw

**Supplementary Table S1 |** The adsorption energies ($E_{ads}$, given in eV) of atomic oxygen (O*) and hydroxy radical (OH) on different adsorption sites corresponding to the model catalysts of the Pd(111) benchmark, $Ni_1$, $Ni_2$, $Ni_3$, $Ni_4$, $Ni_7$, $Ni_{10}$, $Ni_{13}$, $Ni_{1Ml}$, and $Ni_{2ML}$.

| | O*-site | $E_{ads}$ | OH-site | $E_{ads}$ |
|---|---|---|---|---|
| **Pd(111)** | Pd-h | −1.22 | Pd-t | −2.35 |
| | Pd-f | −1.41 | Pd-b | −2.62 |
| **$Ni_1$** | Ni-f | −1.42 | Pd-t1 | −2.45 |
| | Pd-h1 | −1.30 | Pd-t2 | −2.46 |
| | Pd-h2 | −1.29 | Pd-t3 | −2.44 |
| | Pd-f1 | −1.42 | Pd-b1 | −2.73 |
| | | | Pd-b2 | −2.75 |
| | | | Pd-b3 | −2.74 |
| **$Ni_2$** | Ni-f | −1.42 | Pd-t1 | −2.45 |
| | Pd-h1 | −1.29 | Pd-t2 | −2.47 |
| | Pd-h2 | −1.28 | Pd-t3 | −2.46 |
| | Pd-f1 | −1.42 | Pd-b1 | −2.76 |
| | | | Pd-b2 | −2.77 |
| | | | Pd-b3 | −2.75 |
| **$Ni_3$** | Ni-h | −1.25 | Pd-t1 | −2.45 |
| | Ni-f | −1.40 | Pd-t2 | −2.48 |
| | Pd-h1 | −1.25 | Pd-t3 | −2.47 |
| | Pd-h2 | −1.30 | Pd-b1 | −2.75 |
| | Pd-f1 | −1.40 | Pd-b2 | −2.78 |
| | | | Pd-b3 | −2.76 |
| **$Ni_4$** | Ni-h | −1.26 | Pd-t1 | −2.46 |
| | Ni-f | −1.40 | Pd-t2 | −2.44 |
| | Pd-h1 | −1.24 | Pd-t3 | −2.45 |
| | Pd-h2 | −1.30 | Pd-b1 | −2.75 |
| | Pd-f1 | −1.35 | Pd-b2 | −2.75 |
| | Pd-f2 | −1.43 | Pd-b3 | −2.64 |
| **$Ni_7$** | Ni-h | −1.18 | Pd-t1 | −2.43 |
| | Ni-f1 | −1.38 | Pd-t2 | −2.46 |
| | Ni-f2 | −1.35 | Pd-t3 | −2.47 |
| | Pd-h1 | −1.23 | Pd-b1 | −2.70 |
| | Pd-h2 | −1.30 | Pd-b2 | −2.74 |
| | Pd-f1 | −1.41 | Pd-b3 | −2.75 |
| | Pd-f2 | −1.47 | | |

| | O*-site | $E_{ads}$ | OH-site | $E_{ads}$ |
|---|---|---|---|---|
| **$Ni_{10}$** | Ni-h | −1.14 | Pd-t1 | −2.39 |
| | Ni-f1 | −1.31 | Pd-t2 | −2.38 |
| | Ni-f2 | −1.32 | Pd-t3 | −2.42 |
| | Pd-h1 | −1.19 | Pd-b1 | −2.72 |
| | Pd-h2 | −1.27 | Pd-b2 | −2.66 |
| | Pd-f1 | −1.34 | Pd-b3 | −2.62 |
| | Pd-f2 | −1.33 | | |
| **$Ni_{13}$** | Ni-h | −2.23 | Ni-t1 | −3.11 |
| | Ni-f1 | −2.77 | Pd-t1 | −2.43 |
| | Ni-f2 | −1.90 | Pd-t2 | −2.87 |
| | Pd-h1 | −1.81 | Ni-b1 | −3.43 |
| | Pd-h2 | −1.39 | NiPd-b1 | −3.11 |
| | Pd-f1 | −1.46 | Pd-b1 | −2.71 |
| | Pd-f2 | −1.45 | | |
| **$Ni_{1ML}$** | Pd-h | −1.21 | Pd-t | −2.40 |
| | Ni-f | −1.35 | Pd-b | −2.67 |
| **$Ni_{2ML}$** | Ni-h | −1.02 | Pd-t | −2.37 |
| | Ni-f | −1.14 | Pd-b | −2.65 |

**Supplementary Table S2 |** The calculated d-band center ($\varepsilon_d$, unit in eV) at the selected triatomic M-hcp sites on of the Pd(111), $Ni_1$ to $Ni_4$, $Ni_7$, $Ni_{10}$, $Ni_{13}$, $Ni_{1Ml}$, and $Ni_{2ML}$ models corresponding to Figure 2.

| | **Pd** | **$Ni_1$** | **$Ni_2$** | **$Ni_3$** | **$Ni_4$** | **$Ni_7$** | **$Ni_{10}$** | **$Ni_{13}$** | **$Ni_{1ML}$** | **$Ni_{2ML}$** |
|---|---|---|---|---|---|---|---|---|---|---|
| $\varepsilon_d$ | –1.77 | –2.32 | –2.36 | –2.42 | –2.39 | –2.44 | –2.44 | –1.90 | –2.28 | –2.46 |
| *site* | Pd-hcp | Pd-h1 | Pd-h1 | Ni-hcp | Ni-hcp | Ni-hcp | Ni-hcp | Ni-hcp | Pd-hcp | Ni-hcp |

**Supplementary Table S3 |** Reaction energy barrier (ΔE, given in eV) of the selected $O_2$ dissociation (ΔE1) and O* hydrogenation (ΔE2) pathways on the Pd(111) and $Ni_1$ to $Ni_{13}$ models corresponding to Fig. 5(e).

| | **Pd** | **$Ni_1$** | **$Ni_2$** | **$Ni_3$** | **$Ni_4$** | **Ni7** | **$Ni_{1ML}$** | **Ni10** | **$Ni_{2ML}$** | **Ni13** |
|---|---|---|---|---|---|---|---|---|---|---|
| Δ*E*1 | 0.62 | 0.49 | 0.50 | 0.53 | 0.53 | 0.56 | 0.57 | 0.59 | 0.67 | 0.06 |
| Δ*E*2 | 0.21 | 0.30 | 0.31 | 0.25 | 0.24 | 0.29 | 0.34 | 0.28 | 0.33 | 0.40 |

**Supplementary Table S4 |** The adsorption energies ($E_{ads}$, unit in eV) of atomic oxygen (O*) and hydroxy radical (OH) on different adsorption sites corresponding to the model catalysts of the three "Ni1" systems, i.e., $Ni^{SA}$-3rd, $Ni^{SA}$-2nd, and $Ni^{SA}$-1st.

| | O*-site | $E_{ads}$ | OH-site | $E_{ads}$ |
|---|---|---|---|---|
| **$Ni_1$** | | | Pd-t1 | −2.45 |
| | Pd-h1 | −1.30 | Pd-t2 | −2.46 |
| | Pd-h2 | −1.29 | Pd-t3 | −2.44 |
| | Ni-f | −1.42 | Pd-b1 | −2.73 |
| | Pd-f1 | −1.42 | Pd-b2 | −2.75 |
| | | | Pd-b3 | −2.74 |
| **$Ni_2$** | | | Pd-t1 | −2.44 |
| | Ni-h | −1.24 | Pd-t2 | −2.45 |
| | Pd-h2 | −1.27 | Pd-t3 | −2.46 |
| | Pd-f1 | −1.41 | Pd-b1 | −2.74 |
| | Pd-f2 | −1.43 | Pd-b2 | −2.73 |
| | | | Pd-b3 | −2.73 |
| **$Ni_3$** | | | Pd-t1 | −2.81 |
| | Pd-h1 | −1.32 | Pd-t2 | −2.38 |
| | Pd-h2 | −1.33 | Pd-t3 | −2.47 |
| | Pd-f1 | −1.97 | Pd-b1 | −3.12 |
| | Pd-f2 | −1.46 | Pd-b2 | −2.79 |
| | | | Pd-b3 | −2.75 |

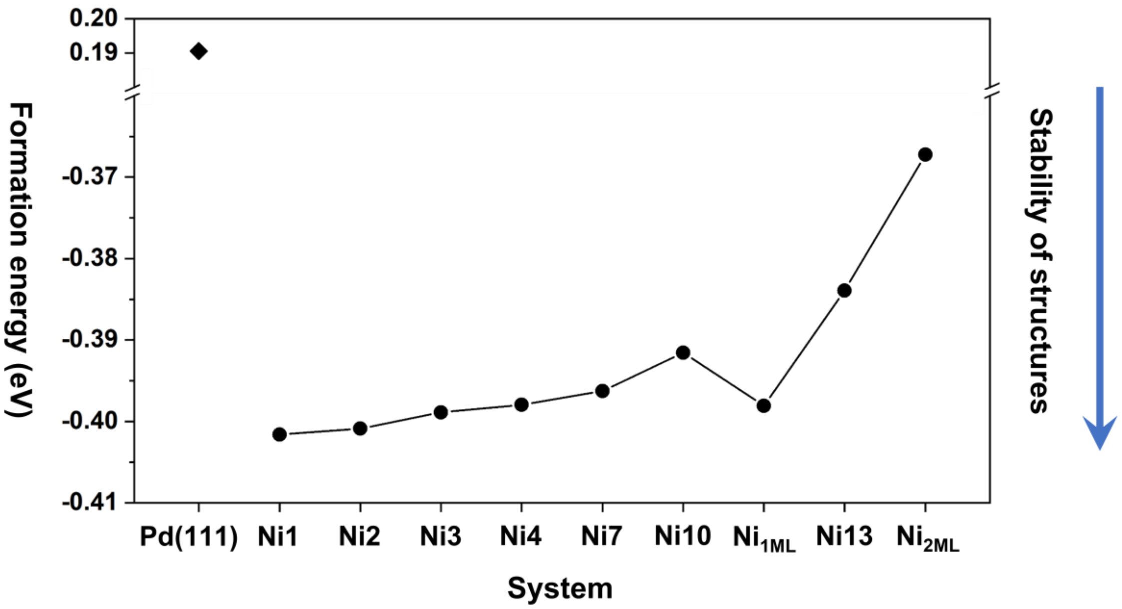


**Supplementary Fig. S1 |** The calculated $E_f$ of all the proposed slabs, including the Pd(111), $NiO_2/Ni^1/Pd$, $NiO_2/Ni^2/Pd$, $NiO_2/Ni^3/Pd$, $NiO_2/Ni^4/Pd$, $NiO_2/Ni^7/Pd$, $NiO_2/Ni^{10}/Pd$, $NiO_2/Ni^{1ML}/Pd$, $NiO_2/Ni^{13}/Pd$ and $NiO_2/Ni^{2ML}/Pd$ systems.

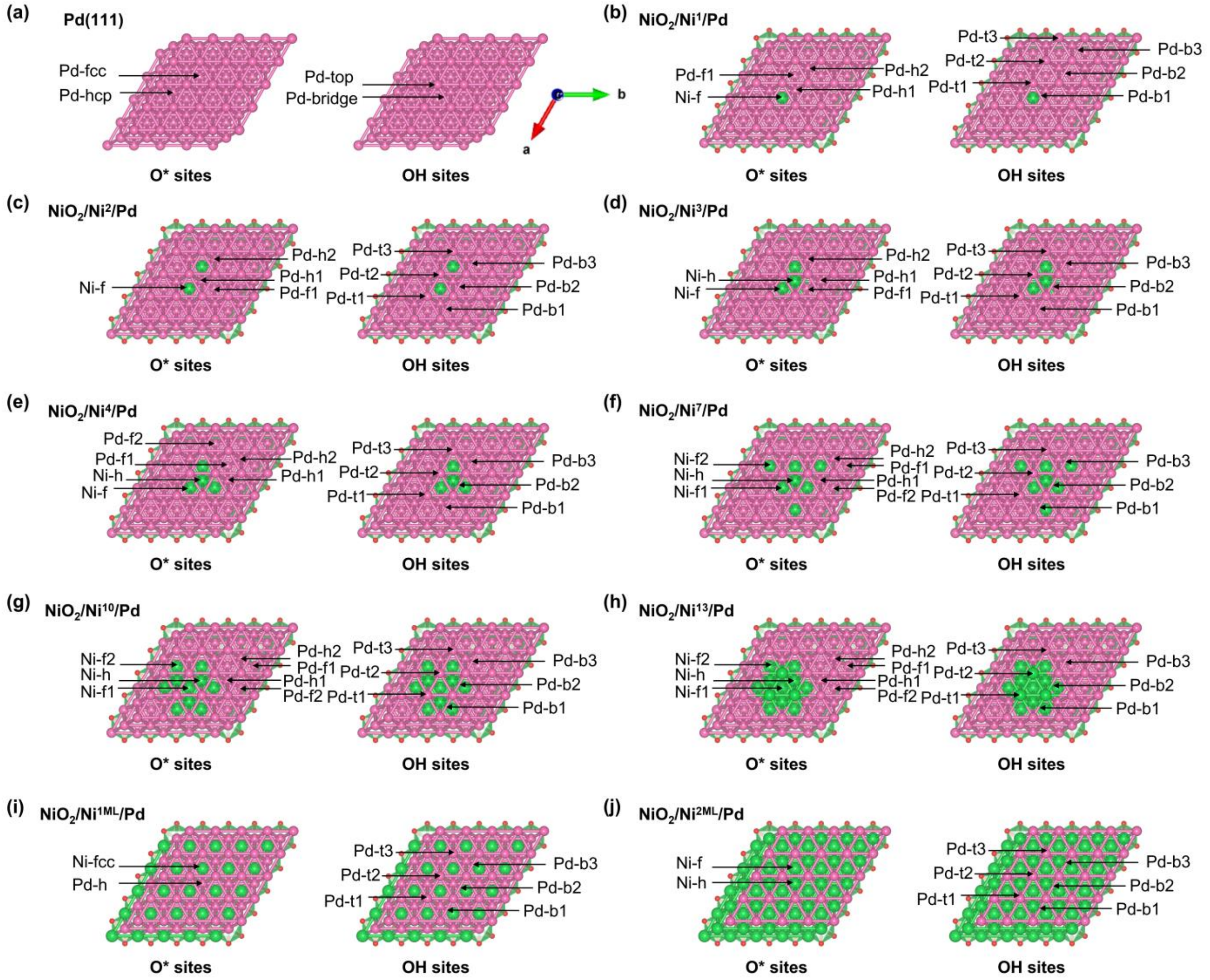


**Supplementary Fig. S2 |** Top views of the atomic adsorption structures of the $O_2$ (left) and O* (right) on (a) pure Pd(111), (b) $NiO_2/Ni^1/Pd$, (c) $NiO_2/Ni^2/Pd$, (d) $NiO_2/Ni^3/Pd$, (e) $NiO_2/Ni^4/Pd$, (f) $NiO_2/Ni^7/Pd$, (g) $NiO_2/Ni^{10}/Pd$, (h) $NiO_2/Ni^{13}/Pd$, (i) $NiO_2/Ni^{1ML}/Pd$ and (j) $NiO_2/Ni^{2ML}/Pd$ surface models. The corresponding data is listed in Table S1. The pink, green and red spheres represent Pd, Ni and O atoms, respectively.

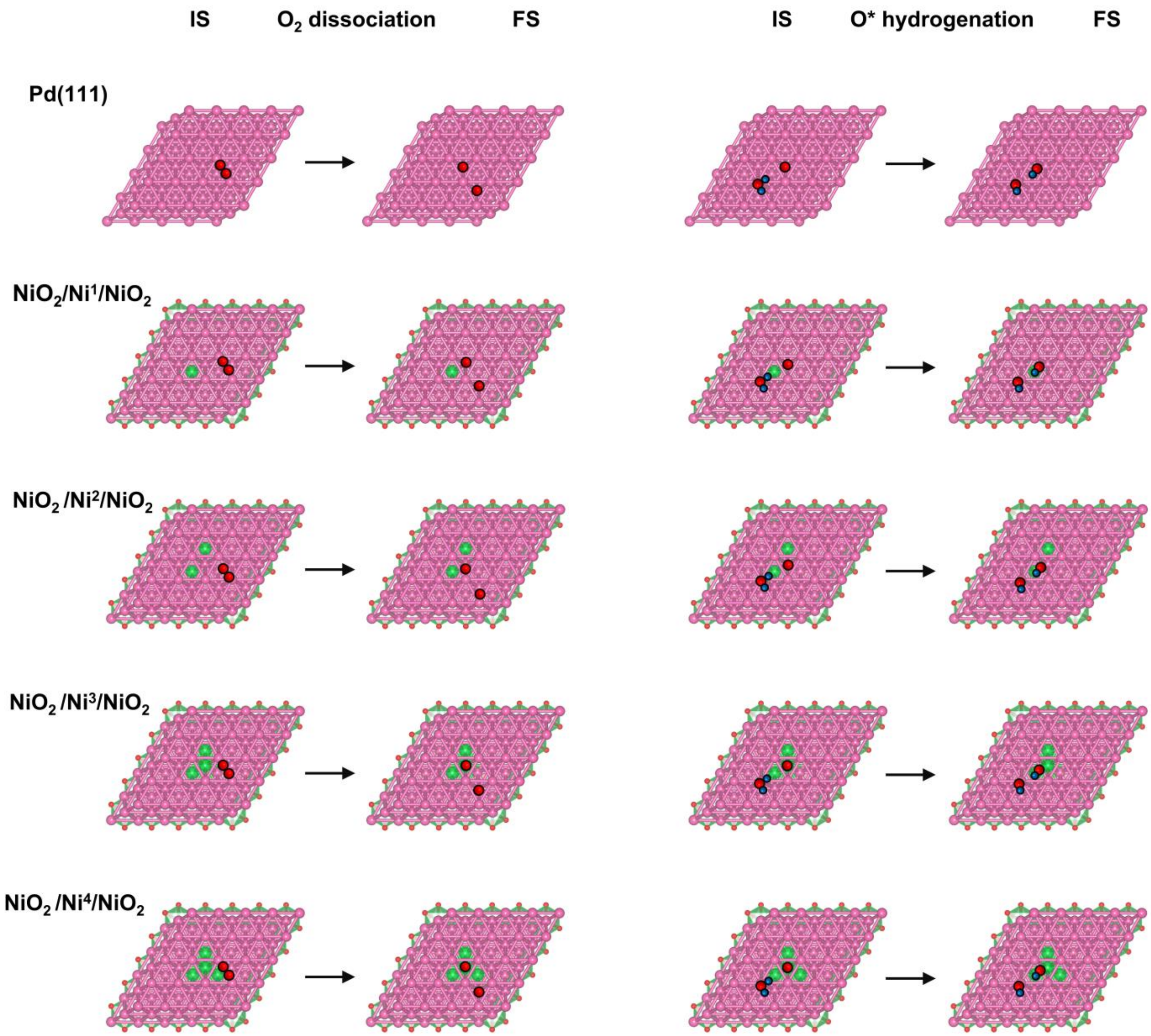


**Supplementary Fig. S3 |** Top view structures of selected $O_2$ dissociation and O* hydrogenation steps in ORR on various surface models, including Pd(111), $NiO_2/Ni^1/Pd$, $NiO_2/Ni^2/Pd$, $NiO_2/Ni^3/Pd$, and $NiO_2/Ni^4/Pd$. The IS and FS respectively stands for the initial-state and final-state. The pink, green and red spheres represent Pd, Ni and O atoms, respectively.

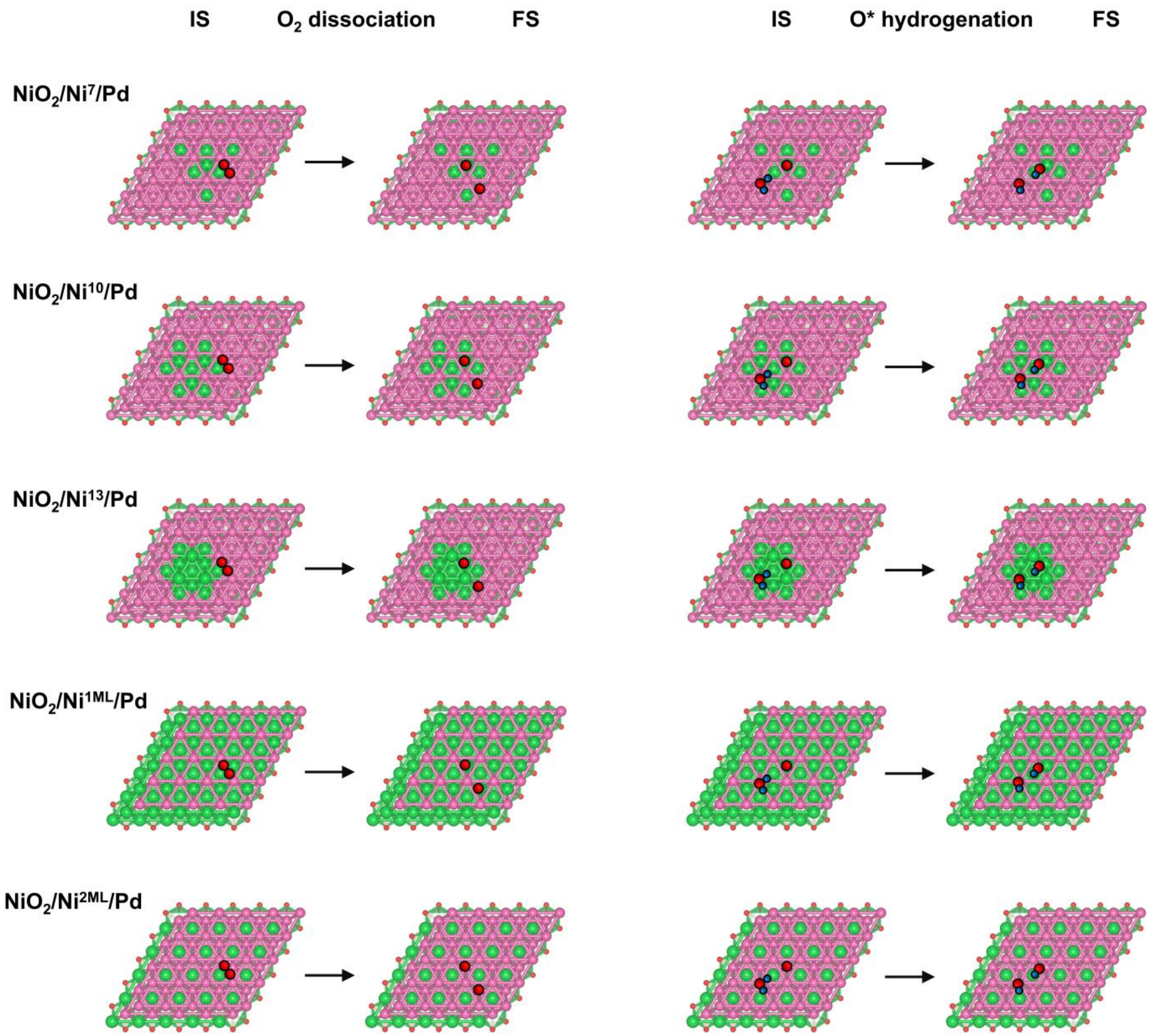


**Supplementary Fig. S4 |** Top view structures of selected $O_2$ dissociation and O* hydrogenation steps in ORR on various surface models, including $NiO_2/Ni^7/Pd$, $NiO_2/Ni^{10}/Pd$, $NiO_2/Ni^{13}/Pd$, $NiO_2/Ni^{1ML}/Pd$ and $NiO_2/Ni^{2ML}/Pd$. The IS and FS respectively stands for the initial-state and final-state. The pink, green and red spheres represent Pd, Ni and O atoms, respectively.

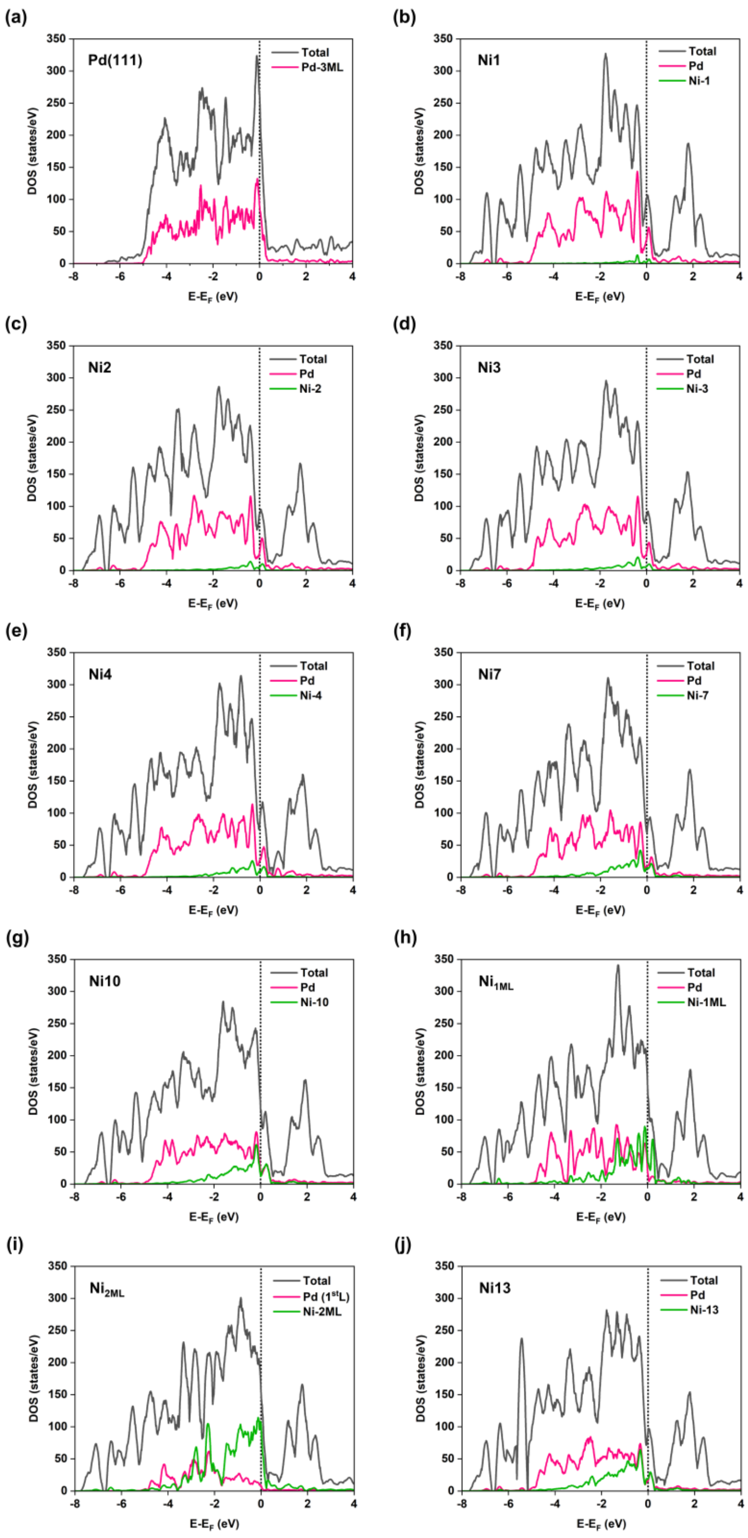


**Supplementary Fig. S5 |** Projected density of states (PDOS) for the surface Pd-layer and the Ni atoms intercalated in the Pd-layer of $NiO_2/Ni^n/Pd$ and reference Pd(111) surface models. The total DOS (TDOS) is projected onto all orbitals of total atoms that constitute the model catalyst, while the PDOS is projected onto the d orbitals of the involved atoms.

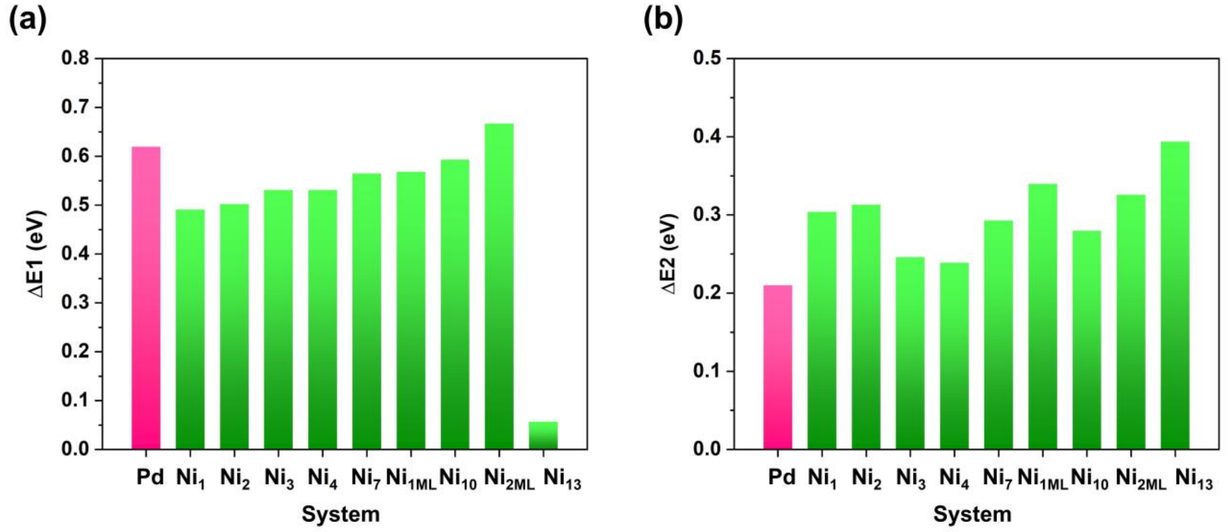


**Supplementary Fig. S6 |** Calculated reaction energy barriers height of (a) $O_2$ dissociation sub-step ($\Delta$E1, eV) and (b) O* hydrogenation sub-step ($\Delta$E2, eV) on the proposed model catalysts in this work.

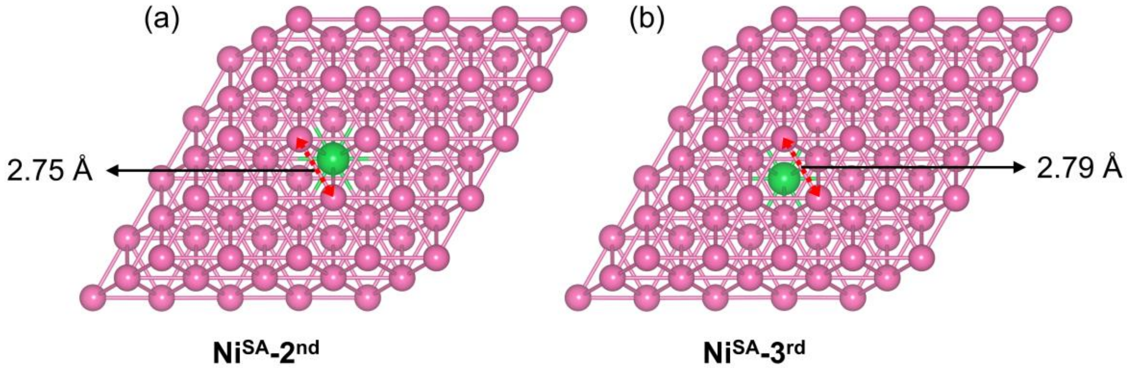


**Supplementary Fig. S7 |** Calculated Pd-Pd bond length of the surface triatomic Pd-hcp site above the doped SA-Ni of the $Ni_{SA}$-2$^{nd}$ and $Ni_{SA}$-3$^{rd}$ systems.

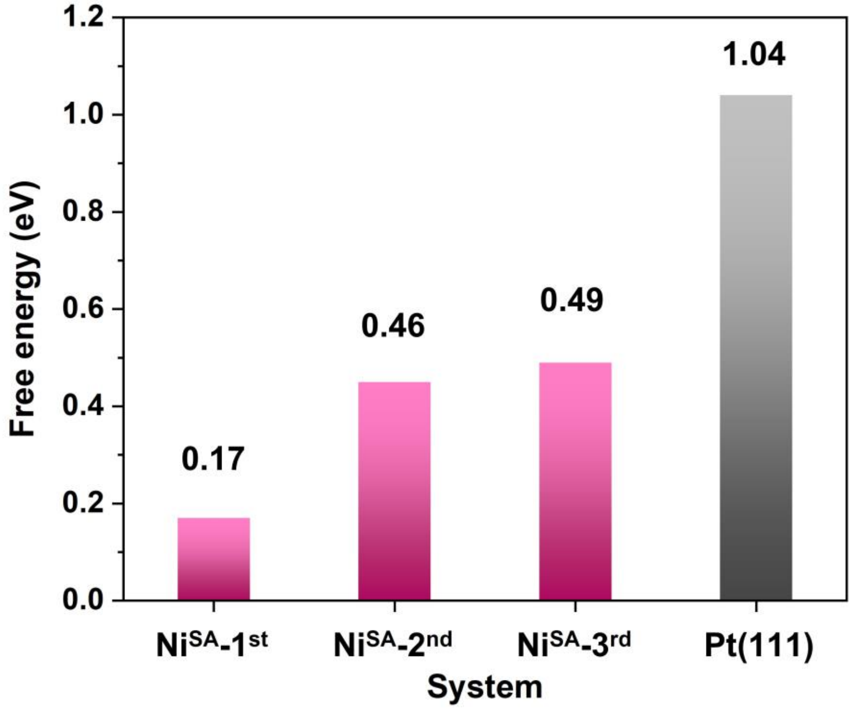


**Supplementary Fig. S8 |** Calculated reaction energy barriers height (ΔE) of the RDS ($O_2$ dissociation step) on the proposed three $Ni^1$ systems and the benchmarking and Pt(111).

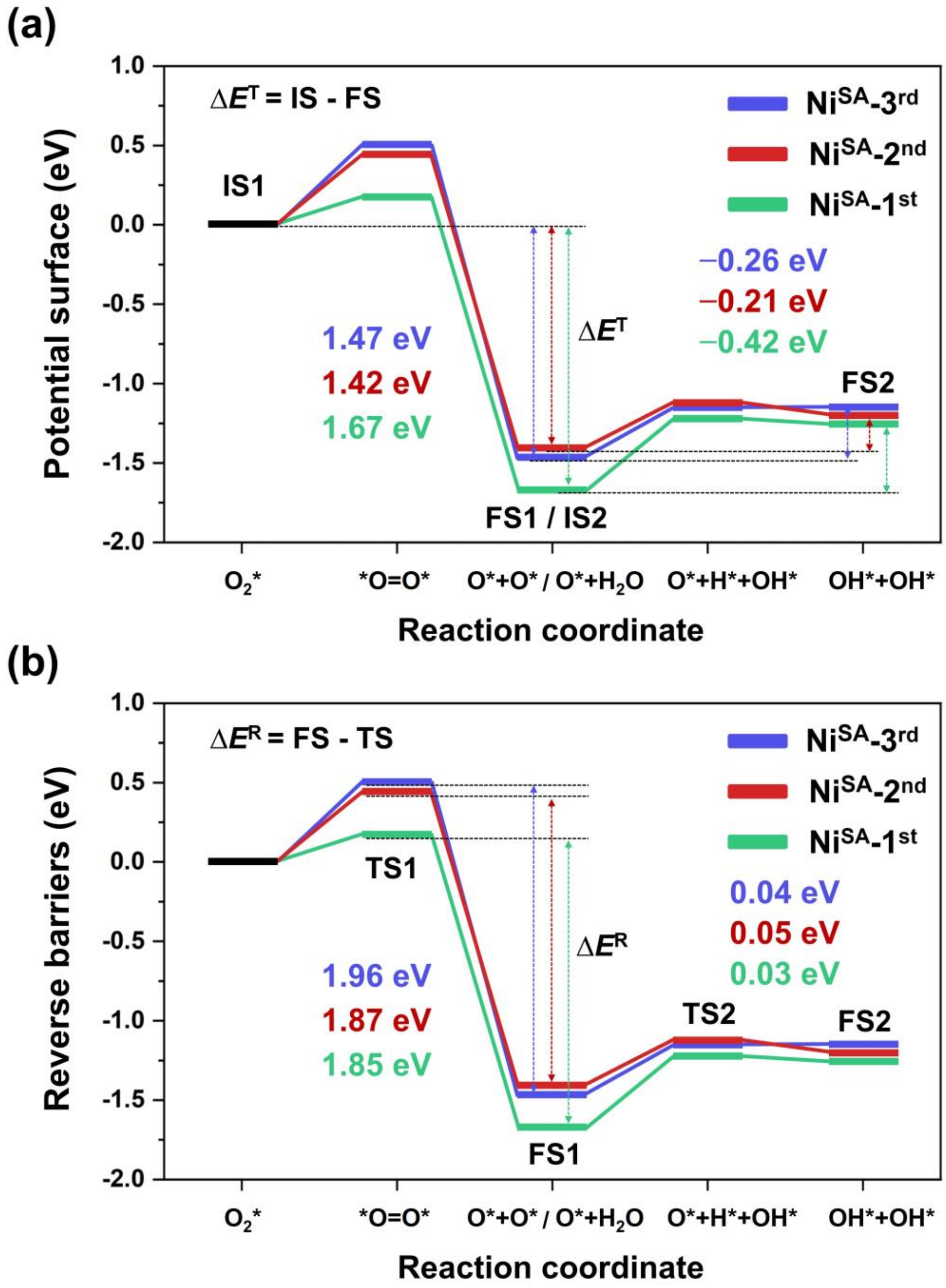


**Supplementary Fig. S9 |** Free energy diagram of a consecutive two-step reaction pathway on the proposed three model surfaces. (a) Thermodynamic potential energy surface ($\Delta E^{T}$) of $O_2$ dissociation ($\Delta E^{T}1$) and O* hydrogenation ($\Delta E^{T}2$) along the complete ORR paths on the three catalysts. (b) Reverse reaction barriers of $O_2$ dissociation ($\Delta E^{R}1$) and O* hydrogenation ($\Delta E^{R}2$) along the complete ORR paths on the three catalysts.

**Supplementary Note S1 | Detailed descriptions and explanations concerning the structural designs and the patterns and positions of the doped Ni atom(s) for the nine proposed model catalysts.**

As illustrated in Fig. 1 (a), the benchmarking Pd(111) surface model composed of six layers of Pd atoms is labeled as the reference group. In Fig. 1 (b) to (c), the Ni atom(s) are intermixed in the 3rd layer of the Pd atoms at the $NiO_2$/Pd interface for the $NiO_2/Ni^1$/Pd and $NiO_2/Ni^2$/Pd surface models, respectively. Fig. 1 (d) and (e) show that three and four Ni atoms are intermixed in the 3rd layer of the Pd atoms shaping into an upright-like trigon and a pyramid-like tetragon for the $NiO_2/Ni^3$/Pd and $NiO_2/Ni^4$/Pd models, respectively. Regarding the $NiO_2/Ni^7$/Pd model, six Ni atoms are doped in the 3rd Pd-layer to shape into a large trigon and one Ni atom is in the 2nd Pd-layer above the center of Ni trigon, as shown in Fig. 1 (f). While, for the $NiO_2/Ni^{10}$/Pd model, seven Ni atoms are doped in the 3rd Pd-layer to shape into a large hexagon and the three Ni atoms are in the 2nd Pd-layer as a trigon above the center of Ni hexagon, see Fig. 1 (g). As regards the $NiO_2/Ni^{13}$/Pd model in Fig. 1 (h), seven Ni atoms are in the 3rd Pd-layer forming a hexagon, three Ni are doped in the 2nd Pd-layer as a trigon above the center of the Ni hexagon, the remaining three Ni atoms are incorporated into the 1st Pd-layer to constitute another trigon right above the Ni trigon in the 2nd Pd-layer, i.e., the $NiO_2/Ni^{13}$/Pd catalyst is the only model with three Ni atoms occupying (exposed on) the outermost surface. In the cases of the $NiO_2/Ni^{1ML}$/Pd and $NiO_2/Ni^{2ML}$/Pd models in Fig. 1 (i) and (j), the lowest one and two Pd layers are totally substituted by Ni atoms, which are regarded as the one Ni monolayer occupied ($Ni^{1ML}$) and two Ni monolayer occupied ($Ni^{2ML}$) models, respectively.

**Supplementary Note S2 | Selection of stable adsorption sites of chemisorbed atomic O (O*), OH radical, $O_2$ and $H_2O$ molecules for the proposed model catalysts.**

Since the ORR is generally a two-substep electrochemical process, which contains a first absorbed oxygen molecule dissociation ($O_2^* \rightarrow 2O^*$) and the second absorbed atomic oxygen hydrogenation ($O^* + H_2O \rightarrow 2OH$). Hence, the atomic oxygen (O*) is undoubtedly the most pivotal intermediate-product in the ORR, which is generated from the decomposition of the absorbed oxygen molecule in the first $O_2$ splitting substep.

In general, it is always believed that the most stable chemisorption configuration on metal is the *three-fold* hollow sites (i.e. M-h / f site, where h and f stand respectively for the hcp and fcc sites) for atomic O (O*), *one-fold* top / *two-fold* bridge site (i.e. M-t / M-b site) for OH radicals, *two-fold* bridge site (i.e. M-b site) for $O_2$ molecule, and top site (i.e. M-t site) for $H_2O$ molecule on a catalyst surface; where "M" is Pd or Ni atoms in this study.

**Supplementary Note S3 | Detailed discussions about the charge transfer/relocation scenarios and their impact on the ORR activity of $Ni_1$ systems.**

Further, the underlying charge relocation between the interfacial intercalation of $Ni^{SA}$ and its surrounding atoms in the $Ni_1$ system was evaluated by charge density difference and Bader charge calculations on behalf of the $NiO_2/Ni^n$/Pd series. From Fig. 4 (f) and (h), the 3D plots of charge density difference exhibit a significant tendency of charge accumulation congregating between the interfacially

intercalated $Ni^{SA}$ and its surrounding Pd atoms via different perspectives. In particular, the charge redistribution is found to be agglomerated over the doped Ni atom, which directly affects the adsorption properties of the surface triatomic Ni-*fcc* site right above the $Ni^{SA}$ and significantly improves the redox ability of the triatomic site and its neighboring regions, since the nature of the catalysis is the charge exchange between the catalyst and its adsorbates. More explicitly, extra available charge injected to the Ni-*fcc* site is believed can enhance the capture capacity of the electroneutral $O_2$ and $H_2O$ to promote their chemisorption, simultaneously, introduce/tune the repulsive force for the electronegative O* and OH to facilitate their desorption from the catalyst surface, therefore, synergistically boost the two ORR-steps moving forward in the positive direction.

In addition, the Bader charge population was also calculated to quantify the charge transfer of the specific atoms/elements, as shown in Fig. 4 (g), the corresponding specific atoms and locations in the $NiO_2/Ni^1/Pd$ model are marked in Fig. 4 (h). It can be observed that one of the three Pd atoms composed of the triatomic Ni-*fcc* site above the $Ni^{SA}$ (the green bar) possesses an extra charge of 0.03 $e^-$ than the Pd atom in benchmarking pure Pd (the orange bar), while, the interfacially doped single Ni atom and the O atom coordinates with it below (the green bars) are also found to have obtained excess charge of 0.85 $e^-$ and 2.39 $e^-$ compared to the Ni atom and O atom in pure $NiO_2$ (the orange bar), respectively. Consequently, the additional charge obtained to the $Ni^{SA}$ and its above Pd atoms due to the synergistic effect (strain, ligand and geometric effects) enables the surface Ni-*fcc* site a higher catalytic activity compared to pure Pd benchmark, let alone the core-component $NiO_2$ octahedron. Since the essence of alkaline ORR catalysis is a process of four-electron transfer between adsorbates and surface metal atoms, meanwhile, oxygen atoms prefer to obtain a charge to satisfy their stable adsorbed state on a metal surface based on the valence charges balance of octet rule in VSEPR model. Thus, more available excess charge within a local domain of the catalyst surface undoubtedly improves the redox behaviors of O*, i.e., the overall performance of the ORR. Additionally, the large increase in the charge of the O atom coordinated with the $Ni^{SA}$ means a much stronger Ni-O electrovalent bond, i.e., a thermodynamically more stable core-shell doping structure. Despite the same surface atomic arrangement of the $NiO_2/Ni^n/Pd$ systems (except the $Ni_{13}$) as reference Pd(111), the surface charge distribution of the $NiO_2/Ni^n/Pd$ is unbalanced by the interfacially intercalated Ni atom(s)/cluster. Such a phenomenon gives rise to the local domain disparity by changing the chemical identity distribution, thus, the adsorption competition towards the intermediate O* between neighboring hollow sites can be suppressed with effect. These physical phenomena echo and explain the aforementioned changing trends of the $\varepsilon_d$ and PDOS for the models of interest, as well as the chemical appearance of the significantly improved adsorption properties of the key ORR-species (i.e., O*, OH) in the previous section.

**Supplementary Note S4 | Details concerning the stable initial-state (IS), transition-state (TS) and final-state (FS) geometries of the "$O_2$ dissociation" and "O* hydrogenation" steps on the *Fcc* (111) facet of the proposed model catalysts.**

In terms of the atomic structure investigation and selection for the stable IS and FS of the two stages on the proposed surface models: here, for the 1st substep "$O_2$ dissociation", generally, in the first step "$O_2$ dissociation", the chosen initial-state (IS) and final-state (FS) geometries are respectively an $O_2$ molecule adsorbing on a diatomic bridge site and then being split into two atomic O* that relocate onto two adjacent hollow sites. In the second step "the O* hydrogenation", the chosen IS and FS are one adsorbed O* atom on the hollow site interacting with a neighboring $H_2O$ molecule adsorbed on a metal atom. The final product is two OH radicals. The transition-state (TS) structures (the highest NEB image) in the 1st and 2nd step reactions are respectively considered as the moments (i.e., the reaction energy barrier height) of O-O bond breakage and O-H bond formation.

From our calculations, the *three-fold* hollow sites composed of three metallic atoms (i.e., M-hcp and M-fcc sites, M = Pd or Ni) are energetically more favorable for O* landing other than the *one-fold* top or *two-fold* bridge sites for the $NiO_2/Ni^n/Pd$ and Pd(111) surface models with the FCC(111) facet. Atomic structures for the two-step paths on different surfaces of the $NiO_2/Ni^n/Pd$ systems as well as the benchmark Pd(111) are presented in Supplementary Figs. S3 and S4.

**Supplementary Note S5 | Detailed descriptions and discussions involving the variation relations between calculated $E_{ads}$, ΔE and proposed structures of all model catalysts.**

Fig. 6 (a) exhibits the $E_{ads}$-O* at various adsorption sites of all the proposed model catalysts, where a distinctive fitting crescent-shaped trendline for the $E_{ads}$-O* distribution is observed from the pure Pd to $Ni_{13}$ models. Generally speaking, the $E_{ads}$-O* enhances from the Pd benchmark to $Ni_1$ (or $Ni_2$) due to the discrepancy of model configuration and presence of Ni in the Pd-layer, and subsequently increases gradually to the $Ni_{2ML}$ with the doped Ni atoms increase, which roughly follows the sequence: $Ni_1$, $Ni_2$, $Ni_3$, $Ni_4$, $Ni_7$, $Ni_{1ML}$, $Ni_{10}$, $Ni_{2ML}$. Then, the last model $Ni_{13}$ is observed to show an evident discrete-distribution with significantly enhanced $E_{ads}$-O* compared to the rest due to its exclusive configuration of three Ni atoms naked on the outermost Pd surface. Analogical to the $E_{ads}$-O*, the $E_{ads}$-OH from Fig. 6(b) basically reflects a comparable tendency of arc-shaped distribution from pure Pd to $Ni_{13}$, with the $Ni_2$ (or $Ni_3$) as the inflection point. Another aspect, for the impact of the calculated $E_{ads}$-O*/-OH on the simulated ORR kinetics, both of the barrier distributions of the $\Delta E1$ as well as $\Delta E2$ exhibit the conspicuous crescent-typed fitted trendline for the proposed models from pure Pd to $Ni_{13}$, as shown in Figs. 6(c) and (d). Particularly in Fig. 6(c), the variation trend of the RDS's $\Delta E1$ is found to be almost identical to the change tendency of the $E_{ads}$-O*, which well proves our predicted dependency between $\Delta E1$ versus $E_{ads}$-O* mentioned above. As for the $\Delta E2$, it is still observed to feature a roughly semi-arc change tendency in the same sequence from pure Pd to $Ni_{13}$. Though it seems to cause a slightly challenging barrier ($\Delta E2$) on the second stage for $Ni_1$ (0.3 eV), the impact of its barrier increment is considered marginal compared to the first RDS stage within the calculated thermodynamic coordinates of the integrated ORR path mentioned in Section 3.4.
